# Biological relevance of fatty acyl heterogeneity to the neural membrane dynamics of *Rhesus* macaques during normative aging

**DOI:** 10.18632/oncotarget.11190

**Published:** 2016-08-10

**Authors:** Sin Man Lam, Gek Huey Chua, Xiao-Jiang Li, Bing Su, Guanghou Shui

**Affiliations:** ^1^ State Key Laboratory of Molecular Developmental Biology, Institute of Genetics and Developmental Biology, Chinese Academy of Sciences, Beijing, China; ^2^ State Key Laboratory of Genetic Resources and Evolution, Kunming Institute of Zoology, Chinese Academy of Sciences, Kunming, Yunnan, China

**Keywords:** aging, lipidomics, Rhesus macaques, docosahexaenoic acids, polyunsaturated fatty acids, Gerotarget

## Abstract

Lipidomic analyses of the frontal cortex of *Rhesus* macaques across three selected age groups (young, sexually-mature, old) revealed that docosahexaenoic acids (DHAs) displayed notable and unique accretions in sexually-mature macaques for all phospholipid classes examined, which were not observable in all remaining polyunsaturated fatty acids (PUFAs) investigated. On the other hand, arachidonic acid (ARA) exhibited sharp attritions in the membrane lipidomes of sexually-mature macaques, a decline which was attenuated only for cardiolipins (CLs). DHA enrichment in phospholipids was lost in old macaques, with accompanying augmentations in very-long-chain sphingomyelins (VLC-SMs). Age-dependent alterations in membrane lipidomes point to a possibly complex temporal interplay between DHA-enriched membrane microdomains and SM-/cholesterol-rich rafts in neural membranes during normative aging. Lipid co-regulation data revealed an increasingly intense degree of co-regulation between membrane lipid classes with age, and suggest that reduction in CLs during normative brain aging may prompt alternative membrane lipid synthetic pathways driven by a compromised energy availability in the aging brain.

## INTRODUCTION

The brain invests an estimated 26% of its net ATP expenditure in maintaining phospholipid dynamics, which comprises processes including *de novo* biosynthesis, remodeling, as well as preserving the asymmetric distributions of specific types of phospholipids in cellular and subcellular membrane bilayers [1]. Numerous biophysical studies using model membranes have validated that the precise identity of fatty acids attached within a phospholipid molecule represents a major determinant of its membrane properties. For instance, Wassall *et al* has shown that while docosahexaenoic acid (DHA; C22:6) alters the lateral distribution of sterol-enriched raft regions in model membranes, eicosapentaenoic acid (EPA; C20:5), also an omega-3 fatty acid, does not exhibit similar membrane properties [2, 3]. Apart from fatty acid specificity, phospholipid head group *per se* can also give rise to differing membrane properties. Model membrane assays have demonstrated that whereas cholesterol solubility is markedly diminished in phosphatidylcholine (PC) bilayers comprising polyunsaturated fatty acids (PUFAs) at both the *sn-1* and *sn-2* positions, the presence of a single DHA at the *sn-2* position for phosphatidylethanolamine (PE) bilayers is sufficient to elicit similar reduction in cholesterol solubility [4]. A precise distribution of fatty acyl heterogeneity across various phospholipid classes would, therefore, be of immense importance in maintaining membrane lipid integrity and proper functioning of the brain.

A myriad of previous studies based on the technique of gas-chromatography (GC) has cumulatively constructed a rather comprehensive overview of the changing profiles of individual fatty acids in the brain during normative aging and in neurodegenerative diseases [5–7]. While these studies are informative in contributing to our general understanding on the relationship between the overall distribution of individual fatty acids and neurological health, the precise biological meanings of such changes are often masked; as information pertaining to the specific lipid molecules that encompass the different fatty acids is lost in the process of converting the latter to their methylated esters prior to GC analysis. In the current study, an extensive lipid library specific to the brain tissues of *Rhesus* macaques was constructed upon principle of high-performance liquid chromatography coupled with multiple reaction monitoring detection (HPLC-MRM) using electrospray ionization (ESI). ESI is a soft ionization technique that enables the detection of phospholipids, sphingolipids and glycerolipids in their intact forms with minimal loss of structural information, due to the relatively low energy employed in the ionization process [8]. Based on our pilot analysis, the lipid library was further expanded to include fatty acid-specific transitions of individual phospholipid molecules, which in all constitutes more than 700 distinct lipid species, providing an unprecedented view of the changing membrane lipid landscape of the *Rhesus* macaque frontal cortex during the process of normative aging. In addition, it has been estimated that DHA and arachidonic acid (ARA; 20:4), the two PUFAs deemed most critical to brain homeostasis and normal function, are esterified to phospholipids at a ratio of close to 10 000:1 compared to their unesterified free forms [9]. A global profiling of fatty acid compositional changes in each phospholipid class is thus of critical importance to unveiling the precise biological role that individual fatty acid exerts during the process of normative brain aging.

On basis of their high genetic homology to humans (*c.a*. 92.5% to 95%), the *Rhesus* macaques share a remarkably similar profile of normative aging, which include numerous age-related phenotypes such as visual decline, development of cataracts, as well as deterioration in hearing and psychomotor skills [10, 11]. Despite the considerable research efforts dedicated to unraveling the neurological impacts of PUFAs; and that several clinical studies have clearly established a changing pattern of DHA homeostasis during normative aging in humans [7, 12, 13], no clear counterpart has been observed in animal or *in vitro* studies [14]. The lack of a suitable animal model, coupled with the ethical constraints in working with human brain tissues, has largely circumscribed the arena of PUFA brain research. Herein, we report an extensive lipidomic atlas of the changing membrane lipid landscape in the frontal cortex of *Rhesus* macaques during normative aging to details of individual fatty acyls; and shown that the *Rhesus* monkeys displayed a remarkably similar brain lipidomic signature of normative aging to humans with respect to PUFA regulation. Based on the lipid alterations manifested in young through sexually-mature to old monkeys, this study therefore unveils critical gerotargets for brain aging in macaques that could be of potential translational relevance to humans. Correlation matrix analysis was also conducted on the membrane lipidomes obtained from individual age groups in an attempt to unveil the important roles exerted by the complex interplay of membrane dynamics that may essentially underlie the process of normative brain aging,

## RESULTS

### Age-dependent changes in individual lipid classes

**Figure 1:**
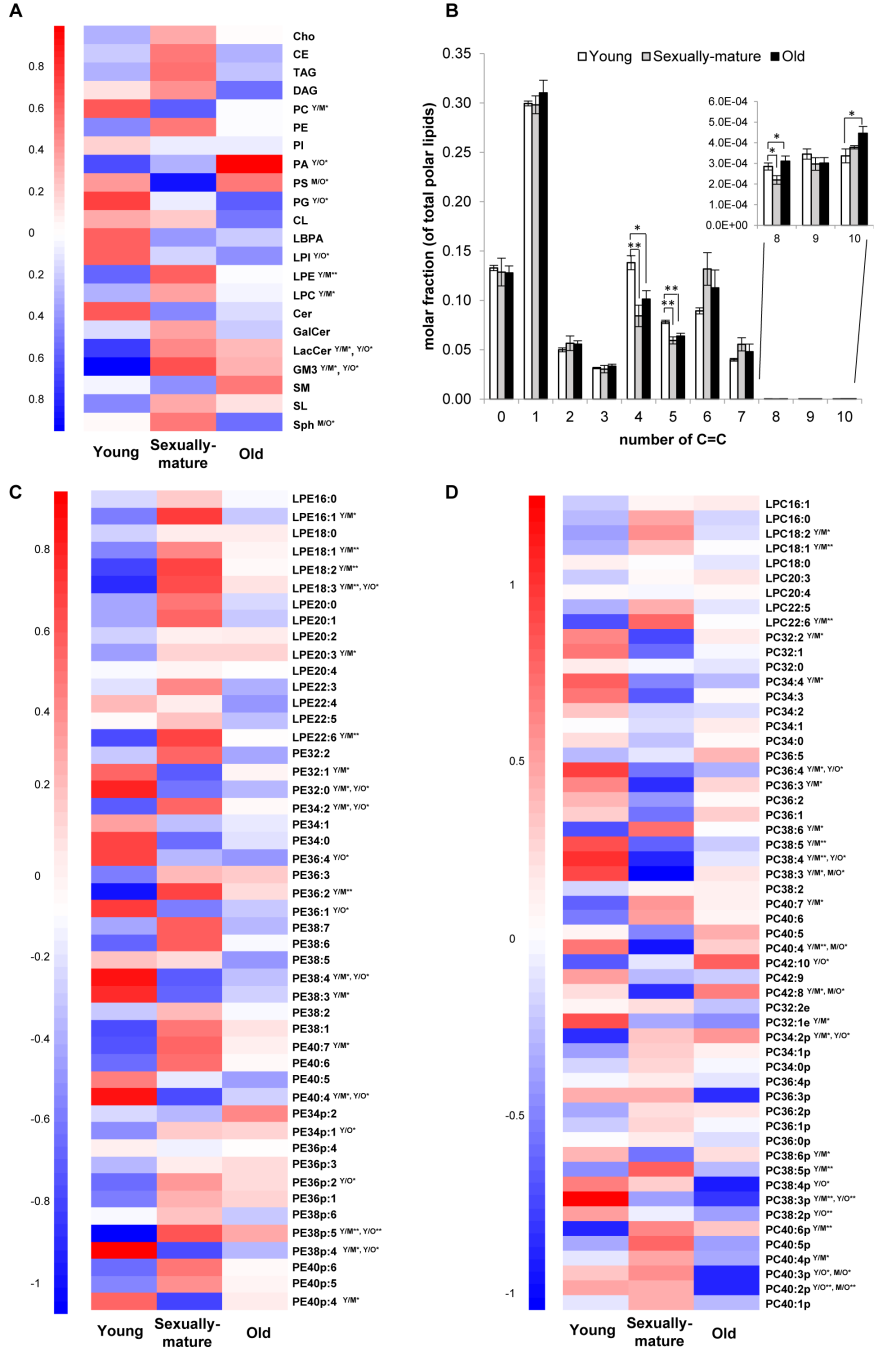
Head group-specific transitions revealed interesting changes in the unsaturation index of membrane lipids across young, sexually-mature and old macaques **A.** Heatmap illustrating changes in 22 individual lipid subclasses. **B.** Alterations in the abundances of lipids comprising varying total double bond number (C = C) in frontal cortical phospholipidome. **C.** Changes in individual LPE and PE species. **D.** Changes in individual LPC and PC species. For heatmaps, the mean of z-scores was plotted for each biological group examined. Y/M denotes comparison between young and sexually-mature monkeys; Y/O denotes comparison between young and old monkeys; M/O denotes comparison between sexually-mature and old monkeys. **p < 0.01; *p < 0.05.

As a preliminary analysis, an analytical library comprising approximately 150 MRM transitions that were specific to the head groups of individual phospholipid species detected in the frontal cortex of *Rhesus* macaques was constructed based on precursor ion scanning. This pilot analytical platform also included an additional 200 sphingolipids and neutral lipids (*i.e*. diacylglycerols DAGs; triacylglycerols TAGs; cholesteryl esters CEs; and free cholesterol Cho); and was employed to analyse the lipidomic changes across the monkeys from the three age-groups (*i.e*. young, sexually-mature and old) under investigation. Age-related changes in the frontal cortex of *Rhesus* macaques for 22 individual lipid classes examined is summarized in Figure 1A. A significant accumulation (*p* < 0.05) of total phosphatidic acids (PAs), the common substrate channeled downstream into the bifurcating pathways for the biosynthesis of zwitterionic phospholipids (Kennedy's pathway) and anionic phospholipids (CDP-DAG pathway) [15],was observed in old monkeys. Accordingly, a significant reduction in total phosphatidylglycerols (PGs) (*p* < 0.05) was noted, which was accompanied by diminished level of total cardiolipins (CLs) downstream, albeit not reaching statistical significance. On the other hand, no significant changes were observed for the levels of zwitterionic phospholipids including PC and PE in old monkeys, while an enhanced level of total phosphatidylserines (PSs) was noted (*p* < 0.05). In addition, total lyso-PC (LPC) and lyso-PE (LPE) were appreciably increased (*p* < 0.05) in sexually-mature monkeys compared to young individuals, with accompanying reduction (*p* < 0.05) in total PC. As for sphingolipids, a general accumulation of glycosylated sphingolipids including lactosylceramides (LacCers) and monosialodihexosylgangliosides (GM3s) were observed (*p* < 0.05) in old macaques, with concomitant reduction (*p* < 0.05) in sphingosine (Sph).

### Age-dependent changes in membrane lipidome according to unsaturation index

An investigation into the unsaturation levels of the frontal cortical phospholipidome revealed decreases in the levels of phospholipids containing four (*p* < 0.05) and five (*p* < 0.01) double bonds in their structures in both sexually-mature and old monkeys compared to their young counterparts (Figure 1B). Interestingly, the relationship between age and unsaturation index in cortical phospholipids does not simply exhibit a linear pattern. Phospholipid species comprising six and seven double bonds were not reduced with age, and slight increases were observed for sexually-mature monkeys indeed, albeit not reaching significance (Figure 1B). These observations suggested fatty acid (or PUFA)-specific changes in cortical phospholipid abundances instead of an overall reduction in PUFAs with age. In line with this, PE species containing six or seven double bonds in their structures, namely PE 38:6, PE 38:7, PE 40:6 and PE 40:7 (*p* < 0.05), were elevated in sexually-mature monkeys; while reductions were observed in species containing four or five double bonds, including PE 38:4 (*p* < 0.05), PE 40:4 (*p* < 0.05) and PE 40:5, compared to young monkeys (Figure 1C). Similar trends were noted for PC, plasmalogen PE (pPE) and plasmalogen PC (pPC) (Figure 1C-1D), despite not all changes observed were statistically significant. As individual parent phospholipid molecule (*i.e*. of a defined number of total carbons and double bonds) can comprise numerous variants each containing different combinations of fatty acids, quantitation of individual phospholipids using head group-specific transitions could mask potentially meaningful age-related changes specific to distinct fatty acids. Therefore, the preliminary analytical method was further expanded to include fatty acid-specific transitions, which expanded the entire lipidomic library to a total of 705 individual lipid species, to monitor fatty acyl-specific changes in cortical membrane lipids of *Rhesus* monkeys during aging.

### Fatty-acyl profile of cardiolipins indicated active remodeling during normative aging

**Figure 2:**
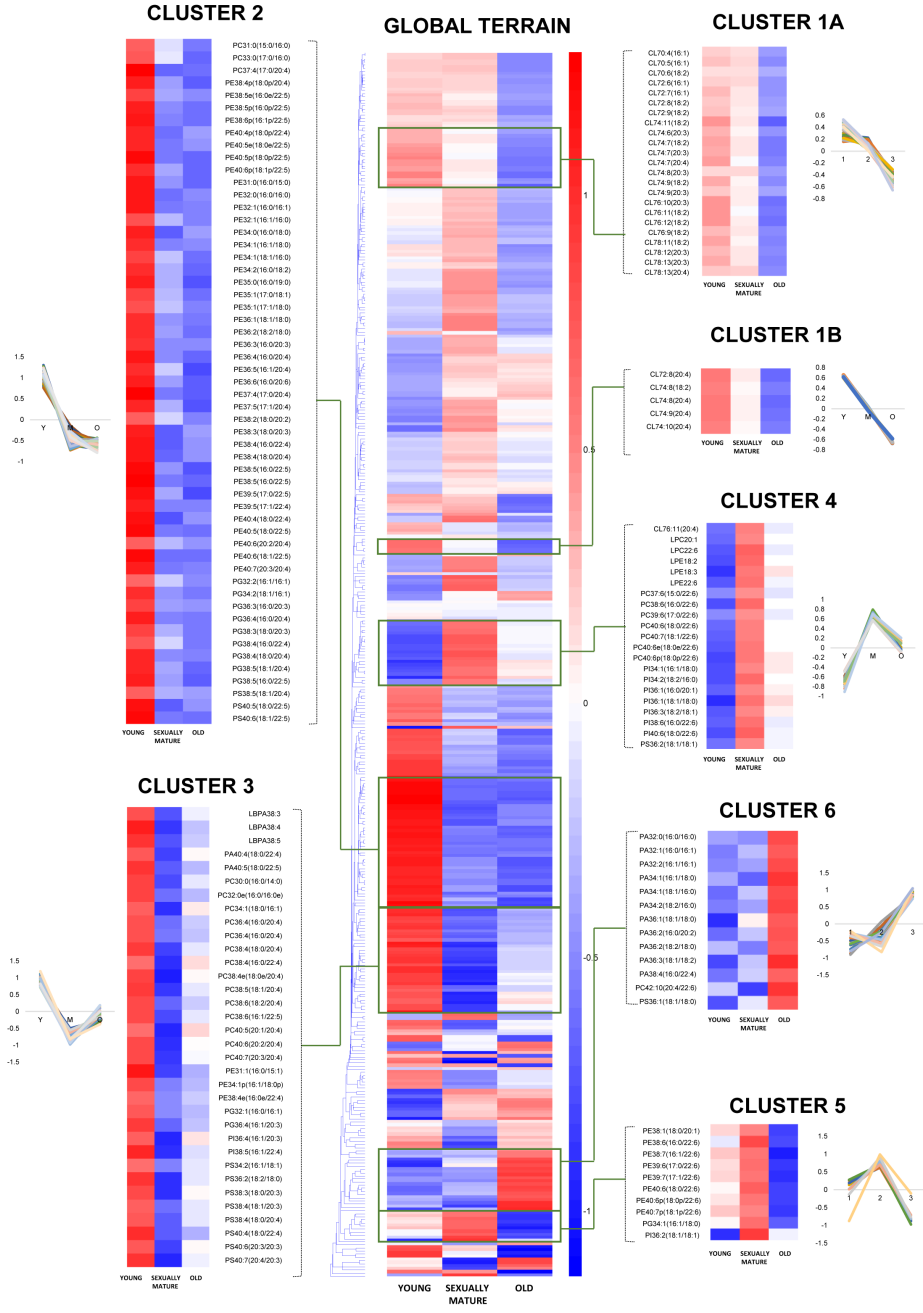
Euclidean clustering of 391 individual phospholipid species detected in the frontal cortex of young, sexually-mature and old monkeys using fatty acid-specific transitions Seven individual clusters with notably distinct patterns of changes across the three age groups were magnified using the same scale as the global terrain map. The mean of z-scores was plotted for each biological group examined.

**Figure 3:**
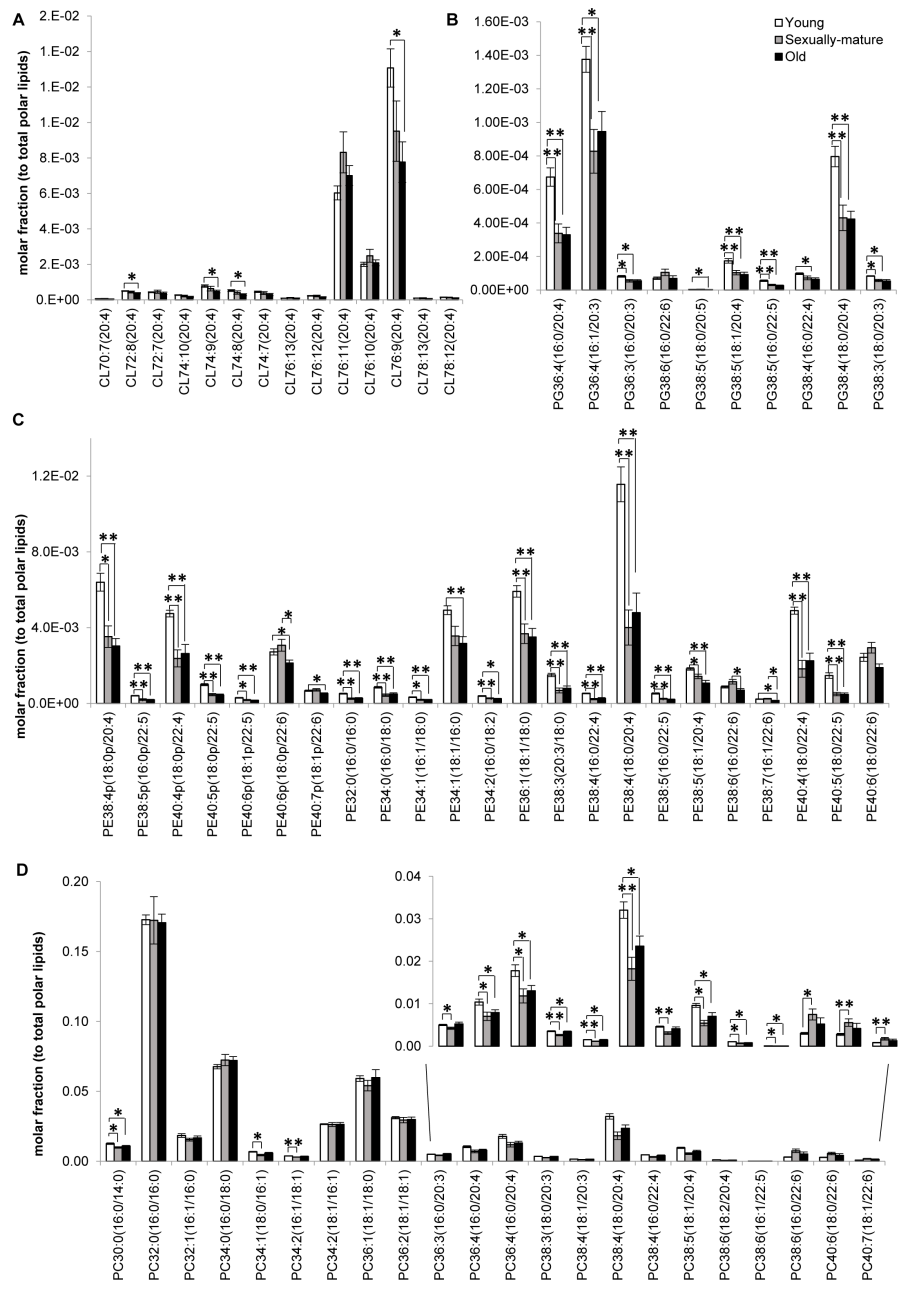
Fatty acid-specific alterations in membrane lipids implicated in cardiolipin biosynthesis and remodeling across young, sexually-mature and old macaques Changes in the levels of individual **A.** cardiolipins, **B.** phosphatidylglycerols, **C.** phosphatidylethanolamines, and **D.** phosphatidylcholines across the three age groups examined were illustrated. Molar fractions normalized to total polar lipids were plotted. **p < 0.01; *p < 0.05.

Our analysis shown that changes in the frontal cortical phospholipidome across the three age-groups obtained using fatty-acyl-specific transitions were consistent with that obtained using head group-based transitions. Furthermore, the expanded method provided a greater resolution in terms of the changes in distinct fatty acyl esterified to individual phospholipids across the three age-groups, and revealed that the increases in species containing a total of six or seven double bonds in sexually-mature macaques (Figure 1B) can be solely attributed to accretions in phospholipids comprising DHAs (see below). A global terrain map obtained by clustering the averaged z-scores of the frontal cortical levels of 391 individual phospholipids across macaques from the three age-groups examined shown that lipids were segregated into distinct clusters, each displaying a unique pattern of change with age (Figure 2). Six meaningful clusters were selected and magnified using the same scale as the global map. Clusters 1A and 1B both contain lipid species that displayed progressive decline in levels with increasing age, with CLs constituting the majority of these clusters (Figure 2). Notably, Cluster 1B contains species predominantly comprising ARA that exhibited a linear reduction with age; while Cluster 1A constitutes CLs of various fatty acids that displayed a more gradual, abated reduction in sexually-mature macaques. CL in the frontal cortex of macaques shown a preponderance of species comprising ARA 20:4 by abundance (Supplemental Figure S1). Notably, the level of CL76:9 (20:4), which by itself constitutes approximately 36% of the total CL levels in young macaques, exhibited a drop of close to 40% in old monkeys compared to young individuals (Figure 3A). CL represent lipids exclusive to the mitochondrial membranes known to be critical for efficient oxidative phosphorylation; by virtue of their abilities in generating membrane bending and creating segregated membrane domains [16, 17]. Thus, the drastic reduction in CL is aligned with an expected decline of mitochondrial metabolic activities with advancing age; and could have serious implications for mitochondrial membrane dynamics.

Accordingly, Cluster 2 comprises several PGs, which serve as immediate substrates for the biosynthesis of CL downstream, that were significantly reduced in both sexually-mature and old macaques compared to young ones (Figure 2). Notably, unlike the trend observed in CL, the reductions in sexually-mature and old monkeys was consistently observed across all PUFA-containing PG except for species containing DHA 22:6; and not only confined to species containing ARA 20:4 (Figure 3B). In addition, species containing ARA 20:4 merely constitute less than 7% of total PG, in contrast to the 30% observed in CL. Since the fatty acid profiles of PG are not directly reflective of that for CL, this suggests that mature CL undergo active remodeling to generate the final acyl chain composition observed in the frontal cortex. In line with this, two other major ARA-containing CL species, namely CL76:11(20:4) and CL76:10(20:4), were not reduced with age, and even slightly elevated in sexually-mature animals compared to young individuals (Figure 3A). Furthermore, CL76:9 (20:4) were significantly reduced only in old monkeys but not in sexually-mature individuals, despite a significant drop in PG precursors in the latter (Figure 3A-3B). These observations cumulatively suggests an active CL remodeling mechanism that serves to tenaciously retain 20:4 (and other PUFAs) in frontal cortical CLs may exist in sexually-mature animals, and to a smaller extent, in old monkeys.

### PUFA-specific changes in membrane lipidome during normative aging

Cluster 2 also constitutes lipid species that shown considerable reductions in both sexually-mature and old monkeys compared to young individuals. This cluster is dominated by polyunsaturated pPE and PE species containing PUFAs in their structures, including C20:3, C20:4, C20:5, C22:4 and C22:5; as well as several saturated/monounsaturated PE species containing fatty acids such as C15:0, C16:0, C16:1, C17:0, C17:1, C18:0 and C18:1 (Figure 2, Figure 3C). Coincidentally, Cluster 3 also comprises several PC, PS, PA and PI species containing similar PUFAs (*i.e*. C20:3, C20:4, C20:5, C22:4 and C22:5) in their structures; which were also significantly and specifically reduced in sexually-mature monkeys and to a smaller extent, in old monkeys compared to young ones (Figure 2; Figure 3D; Figure S2A-S2B). Notably, the reductions in several PUFA-containing phospholipids occurred concurrently in sexually-mature monkeys with the observation that CL, for which PUFAs represent the predominant constituents, were not significantly reduced despite drastic reductions in PUFA-containing PG precursors. Since PEs and PCs represent major constituents of mitochondrial membranes, these classes of lipids may be plausible candidates that serve to confer the PUFA substrates required for CL remodeling based on their physical proximity with mitochondrial CLs in the membrane layers.

Global clustering of lipids revealed an intriguing age-dependent pattern of change in the levels of DHA 22:6 esterified to phospholipids, which was unique amongst PUFAs. Notably, two prominent clusters comprising predominantly of DHA-containing PCs (Cluster 4) and DHA-containing pPEs and PEs (Cluster 5) emerged from global clustering; that both displayed a peak in levels in sexually-mature animals. While DHA-containing PCs were not significantly reduced in old animals (Figure 2 Cluster 4; Figure 3D), noticeable reductions in PEs and pPEs with DHA in their structures were observed in old macaques (Figure 2 Cluster 5; Figure 3C). Interestingly, LPC 22:6 and LPE 22:6 were also segregated into Cluster 4; and their levels were more than doubled in sexually-mature animals compared to young individuals (*p* < 0.01) (Supplemental Figure S4-S5). In addition, while ARA-containing PEs and PCs were significantly reduced (*p* < 0.05) with increasing age (Figure 3C-3D), no corresponding increases were noted for LPE20:4 and LPC20:4 (Supplemental Figure S4-S5); implying that ARA cleaved from these PE and PC species are efficiently transacylated into other membrane lipids in an active remodeling process. In line with this, significant increases were observed for saturated and monounsaturated species including LPE16:1 (*p* < 0.05), LPE18:1(*p* < 0.01), LPC16:0(*p* < 0.05), LPC16:1(*p* < 0.05) and LPC18:1(*p* < 0.05); probably generated as the other remaining products during phospholipid remodeling principally to produce PUFA-containing membrane lipids.

A final cluster of lipids exhibiting appreciable accumulation in old animals is represented by Cluster 6 (Figure 2), which consisted predominantly of PA containing saturated and monounsaturated fatty acids (C16:0, C16:1, C18:0, C18:1). Notably, PS36:1(18:1/18:0), a monounsaturated species found in highest abundance amongst frontal cortical PS, was also segregated into Cluster 6. A closer look into the distribution of individual PA species across the three age groups revealed that the age-dependent accretion was confined to PA species comprising only saturated and/or monounsaturated fatty acids in their structures (Supplemental Figure S2). Namely, major PA species containing PUFA, such as PA38:5(18:1/20:4), PA38:4(18:0/20:4), PA38:3(18:0/20:3), PA40:5(18:0/22:5) and PA40:4(18:0/22:4) did not show appreciable increases in old animals; and were specifically and significantly reduced in sexually-mature animals (*p* < 0.05) instead. Interestingly, DHA-containing PA including PA38:6(16:0/22:6) and PA40:6(18:0:22:6) again did not exhibit similar reduction in sexually-mature animals. A comparable trend was observed for PS compositional alterations across the three age groups (Supplemental Figure S3). Thus, the accretions of total PA and total PS in old monkeys, as shown in Figure 1A, is mainly attributed to increases in species containing saturated and/or monounsaturated fatty acids only, implying an overall increase in the saturation degree of fatty acids in PA and PS in the frontal cortex of old animals.

Remarkably, results derived from our LC/MRM-based analytical approach to monitor fatty acid-specific changes in the frontal cortical phospholipidome of *Rhesus* macaques are in good agreement with a previous report using GC/MS to examine variations in the levels of individual fatty acids in the cerebral cortex of human subjects [7]. Carver *et al* reported a bilinear pattern of variations in PUFA levels in the cerebral cortex, which was divided at the age-point of 18 years in human subjects (corresponding approximately to our sexually-mature age group in *Rhesus* monkeys, see Materials and Methods on *Animals and Tissues*). Specifically, the authors found that PUFA generally sharply decreased with age up to 18 years old, following which the decline is either attenuated or much more gradual; while DHA 22:6 exhibited an increasing trend with a peak in abundance at approximately 18 years, setting itself apart from all remaining PUFAs (C22:5, C22:4, C20:4, C20:3) examined [7]. Therefore, the sharp drop in PUFA-containing phospholipids, with concomitant accumulation of DHA-containing species in sexually-mature macaques seems to recapitulate the age-dependent PUFA alterations in human cerebral cortex.

### Fatty-acyl dependent accumulation of sphingolipids during normative aging

**Figure 4:**
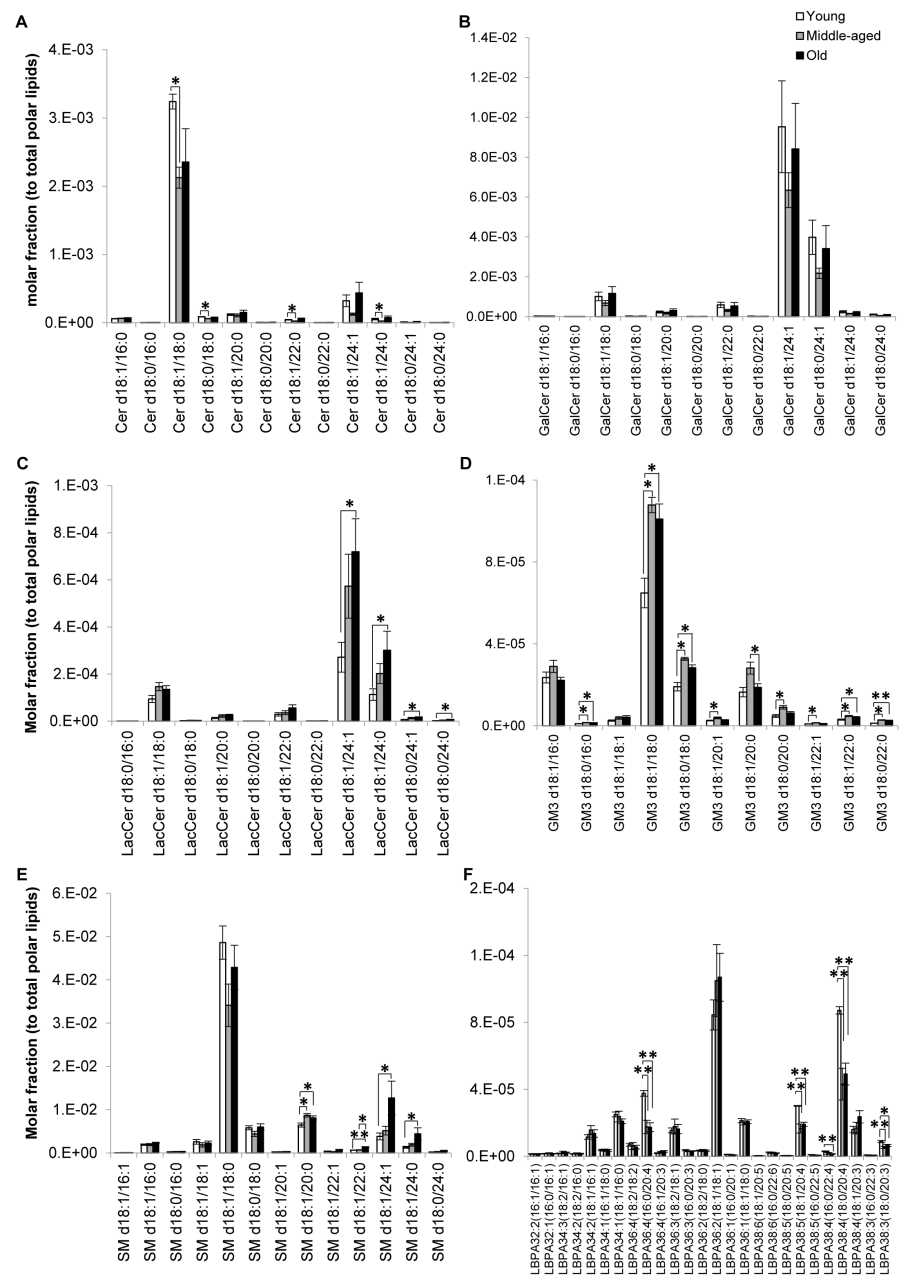
Profiles of sphingolipid and lysobisphosphatidic acid indicated possible aberrations in sphingolipid degradation pathways of old macaques Changes in the levels of individual **A.** ceramides, **B.** galactosylceramides, **C.** lactosylceramides, **D.** monosialodihexosylangliosides, **E.** sphingomyelins and **F.** lysobisphosphatidic acids across the three age groups examined were illustrated. Molar fractions normalized to total polar lipids were plotted. **p < 0.01; *p < 0.05.

Major ceramide (Cer) species were significantly reduced in sexually-mature monkeys irrespective of acyl chain lengths (Figure 4A). On the other hand, no significant differences were noted for galactosylceramides (GalCers) (Figure 4B) and their downstream product sulfatides (SLs) (Supplemental Figure S6), which are almost exclusively synthesized by oligodendrocytes and constitute the major components of myelin sheath [18, 19]. Indeed, major SL species displayed slight but non-significant increases in the older groups, in agreement with previous work that reports a disproportionate degeneration of neocortical regions during aging, with the prefrontal cortical region (examined in the current study) exhibiting selectively prolonged myelination [12]. While both total LacCer (*p* < 0.05) and total GM3 (*p* < 0.05) were appreciably elevated in old monkeys (Figure 1A), compositional profiling indicated that the age-specific increases (*p* < 0.05) were confined to LacCer comprising fatty acids of 24C in length (Figure 4C). On contrary, individual GM3 species were significantly increased particularly in sexually-mature monkeys (*p* < 0.05), and to a smaller extent, in old monkeys (*p* < 0.05) (Figure 4D) in a manner independent of acyl-chain lengths, suggesting that Cer are probably channeled downstream for the biosynthesis of GM3 in sexually-mature monkeys. Remarkably, sphingomyelins (SMs) comprising fatty acids of > C20 were specifically and significantly elevated (*p* < 0.05) in old monkeys (Figure 4E). This observation coincides with reductions in lysobisphosphatidic acids (LBPAs) comprising ARA, most prominently noted for LBPA36:4(16:0/20:4) (*p* < 0.01), LBPA38:5(18:1/20:4) (*p* < 0.05) and LBPA38:4(18:0/20:4) (*p* < 0.01) in old monkeys (Figure 4F).

### Correlation matrix analyses indicated altered patterns of membrane lipid co-regulation during normative aging

**Figure 5:**
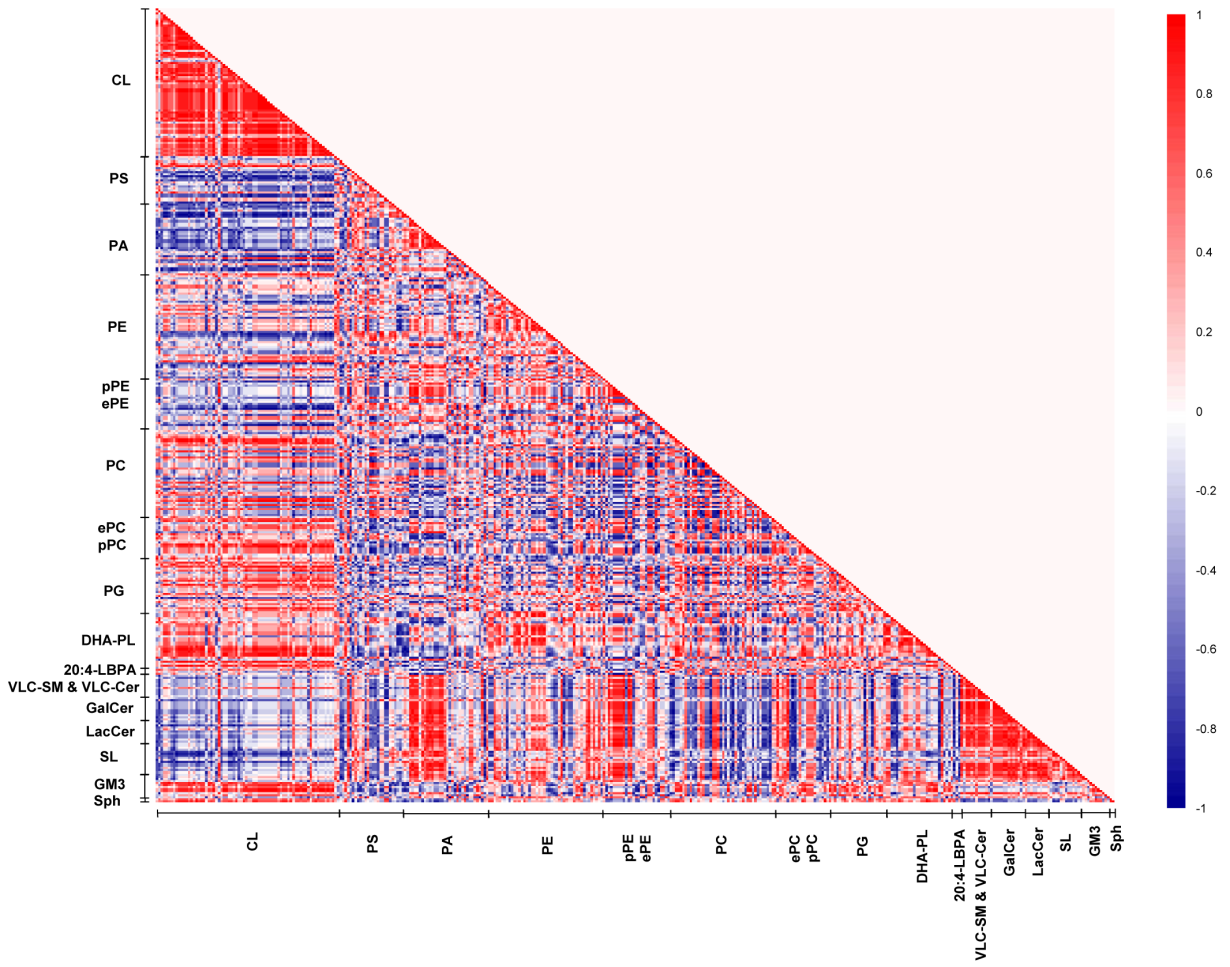
Lipid correlation matrix constructed based on the membrane lipidome of young macaques comprising a selected pool of 386 individual membrane lipid species Vertical axis indicates magnitudes of correlation coefficients (r).

**Figure 6:**
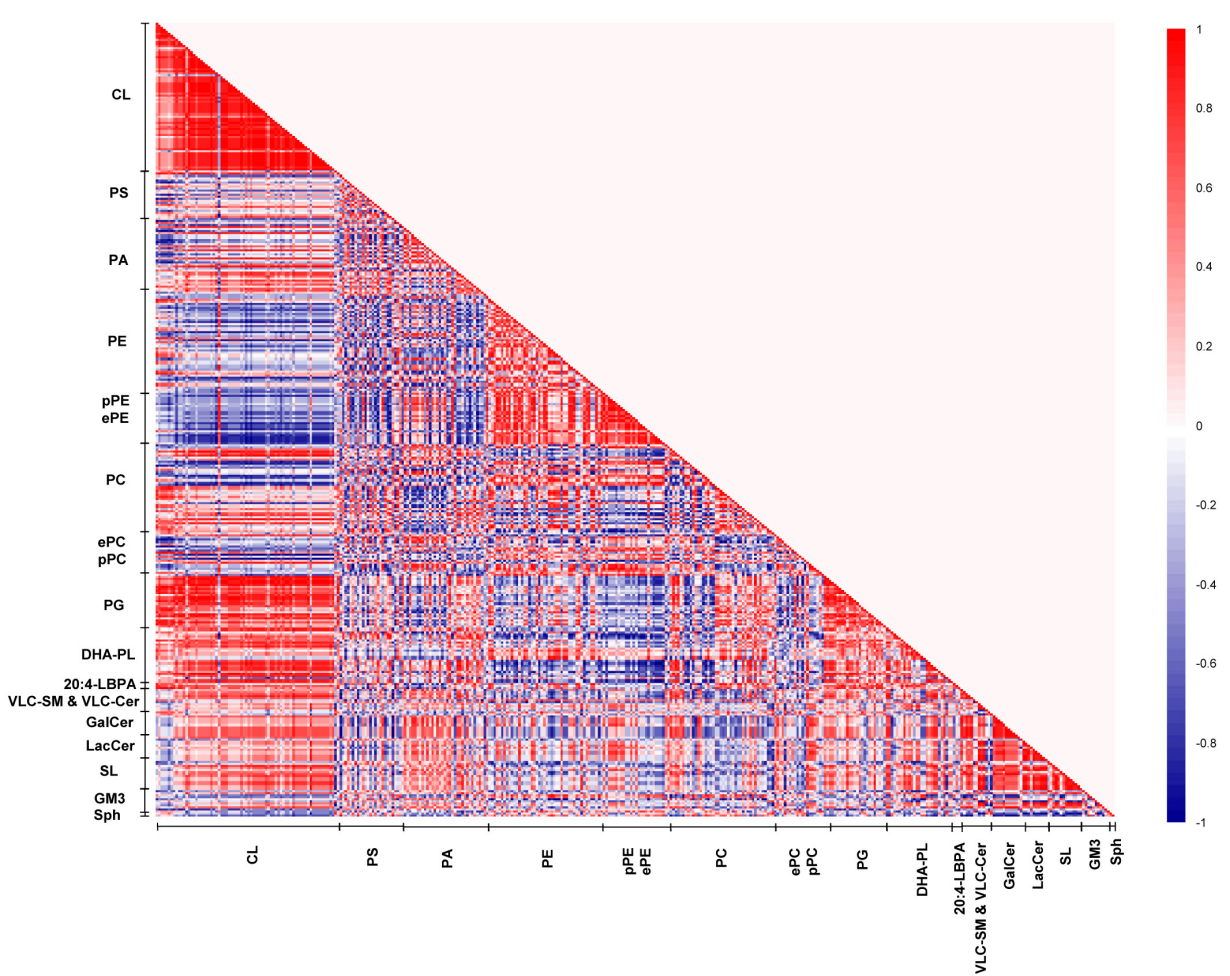
Lipid correlation matrix constructed based on the membrane lipidome of sexually-mature macaques comprising a selected pool of 386 individual membrane lipid species Vertical axis indicates magnitudes of correlation coefficients (r).

**Figure 7:**
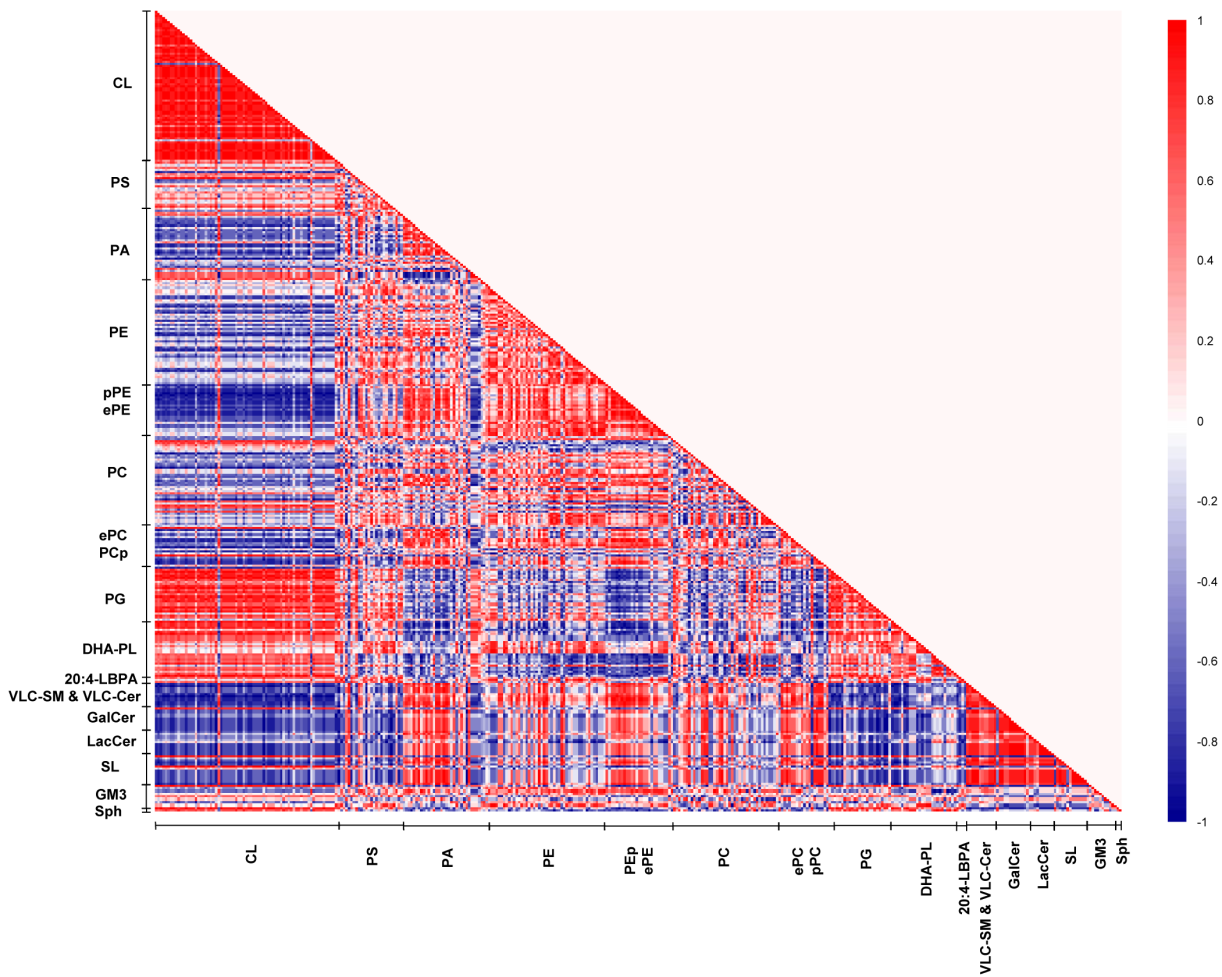
Lipid correlation matrix constructed based on the membrane lipidome of old macaques comprising a selected pool of 386 individual membrane lipid species Vertical axis indicates magnitudes of correlation coefficients (r).

In view of the intriguing patterns of PUFA-specific modifications in membrane lipids across macaques of the three ages, lipid correlation matrix analysis was conducted to unravel possible patterns of age-specific membrane lipid co-regulation that may account for the observed changes. Three distinct lipid correlation matrices were constructed from the frontal cortical membrane lipidomes obtained in young, sexually-mature and old macaques, respectively (Figures 5–7).

In young macaques, DHA-containing PCs displayed strong positive correlations (*r* = 0.6 to 1) with CLs, reaching statistical significance (*p* < 0.05) for species including PC38:6(16:0/22:6), PC39:6(17:0/22:6), PC40:6e(18:0e/22:6) and PC40:6p(18:0p/22:6). On the contrary, saturated/monounsaturated PA species exhibited negative correlations with CLs. For instance, PA32:0(16:0/16:0), PA32:1(16:0/16:1) and PA34:1(16:1/18:0) were negatively correlated (*r* = −0.7 to −1) with several CL species (*p* < 0.05). In addition, DHA-containing PSs and PAs, such as PS40:6(18:0/22:6), PS40:7(18:1/22:6) and PA38:6(16:0/22:6) were negatively correlated (*r* = −0.6 to −1) with VLC-SMs and VLC-Cers, as well as glycosylated sphingolipids including GalCers, LacCers, and SLs; that were approaching significance (*p* < 0.1); while monounsaturated PAs including PA34:1(17:0/17:1), PA36:1(18:0/18:1) and PA36:1(16:0/20:1) were positively correlated (*r* = 0.8 to 1, *p* < 0.05). Thus, in the frontal cortex of young macaques, a relatively high level of DHA-containing phospholipids and concomitantly low level of saturated/monounsaturated PAs are generally associated with enhanced CL accretion; with corresponding diminutions in the levels of VLC-SM, VLC-Cer and glycosylated sphingolipids (Figure 5).

The patterns of lipid co-regulation were strengthened in sexually-mature macaques, as illustrated by distinct clusters of enhanced intensities in the correlation matrix (Figure 6). Notably, the negative correlation between saturated/monounsaturated PAs and CLs in young monkeys was lost in sexually-mature animals. Relatively strong negative correlation (*r* = −0.6 to −0.9) emerged between pPEs and ether PEs (ePEs) containing PUFAs (other than DHA) with major CLs comprising C20:3 and C20:4 in their structures that were approaching significance (*p* < 0.1). The positive correlation between DHA-containing phospholipids and CLs in young macaques was further accentuated in sexually-mature individuals. In particular, two major CL species, namely CL76:11(20:4) and CL76:10(20:4), which displayed slightly augmented levels in sexually-mature individuals, were strongly positively correlated (*r* = 0.7 to 1) with PE39:6(17:0/22:6), PC40:6(18:0/22:6) and PC40:7(18:1/22:6) (*p* < 0.05 for PC; *p* < 0.1 for PE). Based on their elevating proportional abundances in older animals, coupled with accompanying age-dependent reductions in other major CLs comprising 20:4, in particular CL76:9(20:4), these two CL species aforementioned are expected to elicit increasingly important role in mitochondrial functions with advancing age (Supplemental Figure S1). In accordance with their opposite correlations with CLs, a strong negative correlation was observed between phospholipids comprising DHA with ePEs and pPEs containing non-DHA PUFAs. For example, PC38:6(16:0/22:6) was negatively correlated with PE38:4p(16:0p/22:4) (*r* = −0.9; *p* < 0.05), PE40:4p(18:0p/22:4) (*r* = −1; *p* < 0.05), PE36:4e(16:0e/20:4) (*r* = −1; *p* < 0.05), PE38:4e(16:0e/22:4) (*r* = −1; *p* < 0.05) and PE38:5e(16:0e/22:5) (*r* = −1; *p* < 0.05). Therefore, in sexually-mature monkeys, enrichment of DHA-containing membrane lipids is positively associated with increasing level of CLs. In addition, strong negative associations were observed between CLs and PUFA-pPE/ePE species that were absent in young macaques.

On another note, the strong positive correlations observed between LPE22:6 and LPC22:6 with DHA-containing phospholipids, such as PC40:6(18:0/22:6) (*r* = 1; *p* < 0.05) and PC40:7(18:1/22:6) (*r* = 1; *p* < 0.05), indicated that the active uptake of DHAs in the form of lyso-phospholipids across the blood-brain barrier subsequently channeled to active phospholipid remodeling/biosynthesis may at least partially account for the observed accretions of DHA-containing phospholipids in sexually-mature animals. Moreover, the positive correlations between LPE22:6 and LPC22:6 with major CLs such as CL76:11(20:4) (*r* = 1, *p* = 0.02) and CL76:10(20:4) (*r* = 0.9, *p* = 0.08) suggest that the increasing cortical levels of DHA-containing lyso-phospholipids in sexually-mature macaques leads to an accrual of phospholipids comprising DHAs, which may further promote CL biosynthesis/remodeling to maintain a physiologically functional level of CL in this age group.

Remarkably, our correlation matrix analysis revealed that the strongest lipid co-regulation occurs in the frontal cortex of old macaques during normative aging. First, the negative correlations observed between saturated/monounsaturated PAs and CLs in young animals, which were lost in sexually-mature individuals, re-emerged and reached statistical significance in old animals. For example, PA34:1(16:0/18:1), one of the more abundant PA species, is strongly negatively correlated with CL76:9(20:4) (r = −1, *p* < 0.05) in old animals. Remarkably, the negative correlations between pPEs and ePEs with CLs were further augmented in old individuals. In addition, appreciable negative correlations between pPCs and ePCs with CLs emanated in old macaques that were not observed in other age stages. For example, PC40:4e(18:0e/22:4) was negatively correlated with two major CLs in old macaques, namely CL76:10(20:4) (*r* = −1, *p* = 0.02) and CL76:11(20:4) (*r* = −0.9, *p* = 0.08). On another note, while DHA-containing phospholipids remained positively correlated with CLs in old macaques, the intensity of the correlation was attenuated compared to sexually-mature individuals. Unique to old monkeys, strong negative co-regulation was observed between VLC-SMs with DHA-containing PS, PA and PE species, including PS40:6(18:0/22:6) (*r* = −0.7 to −9), PA40:6(18:0/22:6) (*r* = −0.9 to −1) and PE38:6(16:0/22:6) (*r* = −0.7). In addition, LBPA38:4(18:0/20:4) and LBPA36:4(16:0/20:4) were negatively correlated (*r* = −0.8) with VLC-SMs that were approaching significance in old animals.

Despite an increasing degree of saturation was noted in PAs with advancing age (Supplemental Figure S2), primarily due to the accretion in saturated/monounsaturated species with accompanying reductions in polyunsaturated ones, similar alterations were not observed in PCs and PEs further downstream. While no appreciable increases were noted in saturated/monounsaturated PC and PE species, a notable increase (*p* < 0.05) in the monounsaturated PS36:1(18:1/18:0), the most abundant species by abundance, was observed in old macaques (Supplemental Figure S3). Interestingly, appreciable positive correlations were observed between PUFA-PS and PUFA-PE species in old macaques. For instance, PS40:5(18:0/22:5) is strongly positively correlated with PE40:5(18:0/22:5) (*r* = 1, *p* = 0.02); while PS40:4(18:0/22:4) is positively correlated with PE40:4(18:0/22:4) that is approaching significance (*r* = 0.9, *p* = 0.08), indicative of an appreciable level of co-regulation between the levels of PUFA-PS and PUFA-PE in old macaques.

Besides the age-specific lipid co-regulation aforementioned, numerous correlations were consistently observed across all three age groups, such as the positive correlations between PGs and CLs, as well as that between GluCers/GalCers and SLs, which represent well-studied lipid metabolites implicated in common biosynthetic pathways, vindicating the validity and accuracy of the use of correlation matrix analysis for investigating lipid co-regulation in the current study.

## DISCUSSION

Our fatty-acid specific analysis of the monkey frontal cortical membrane lipidome strongly justified the suitability of *Rhesus* macaques as a model for studying normative brain aging in humans, especially with regard to brain PUFA metabolism, since age-dependent PUFA alterations observed in humans in previous works [7, 13] are fully recapitulated in this animal model. Utilization of a fatty acid-specific LC-MRM approach has also unambiguously shown that the intriguing pattern of changes in phospholipid unsaturation index revealed *via* head group-specific transitions can be fully explained by the differential regulation of PUFA esterification in phospholipids with advancing age. Besides, our method based on ESI also confers additional information compared to previous studies employing GC-MS [5, 7] with respect to the specific class of phospholipids that the individual PUFA is esterified to across macaques of different ages.

CLs represent a unique class of phospholipids signature to the mitochondria and are found exclusively in membranes capable of generating an electrochemical gradient ultimately utilized to generate ATPs [16]. Preserving a defined CL compositional profile could be critical in maintaining an optimal level of oxidative phosphorylation in order to sustain energy metabolism of the brain throughout the course of life, as binding of CL to cytochrome c and ADP/ATP carrier had been previously shown to optimize the catalytic functions of these two critical components of the electron transport chain [20, 21]. Our global fatty acid-specific analysis indicated that frontal cortical CLs are capable of maintaining relatively high ARA levels for an extended period of the macaques' lifespan, which is concomitant with corresponding sharp attritions in ARA-containing PE and PC, the major lipid constituents of mitochondrial membranes. This implies that mitochondrial PE and PC could probably supply the ARA substrates required for active CL remodeling in the frontal cortex, although the biological implications of these ARA-containing CLs on brain metabolism warrant further mechanistic investigation. In addition, the strong negative co-regulation observed between CLs and PUFA-pPE/ePE species observed in sexually-mature and old macaques is aligned with our postulation that such species may confer the PUFA substrates required to fuel active CL remodeling. The absence of such correlation in young macaques suggests that CLs are probably maintained principally *via* biosynthesis in the early phase of life, in contrast to remodeling using available phospholipids as substrates later on in life. Indeed, previous works have demonstrated that the fatty acyl compositions of CLs display a high degree of spatial and temporal specificity, and that the precise molecular forms of CL are specially tailored to meet the metabolic requirements of different tissues at varying physiological time-points [16, 22] principally achieved *via* remodeling instead of biosynthesis [16]. It is therefore not surprising that precise phospholipid-CL remodeling mechanism(s) that serves to delicately fine-tune acyl compositions of CLs could exist in the brain, the organ known to possess the highest energy consumption [23], to meet the changing metabolic demands throughout the course of life. In fact, such temporally-specific regulation of CL composition has previously been observed postnatally in mammalian brains, which has been associated with the extensive neuronal-remodeling after birth, giving rise to markedly enhanced levels of ARA and, to a smaller extent, DHA in the postnatal pool of brain CLs [24].

The major functions of PUFAs in the brain can be broadly classified into (1) regulation of membrane dynamics and (2) modulation of cell signaling pathways/neurotransmission *via* conversion into biologically active lipid mediators [9]. Nonetheless, the pool of unesterified PUFAs directed to the biosynthesis of lipid mediators is still relatively minor in abundance compared to the major pool esterified to membrane lipids. Clinical research dedicated to investigating the effects of PUFA on brain aging and neurodegenerative disorders has reached a unanimous consensus in terms of the neurological benefits conferred by an adequate supply of DHA throughout human lifespan; and the cognitive improvement is highly specific to DHA *per se* [25]. A myriad of studies has also previously vindicated the uniquely positive role exerted by DHA in altering neural membranes in a manner that promotes cognitive function and neurological health in general [26–28], which are not observed with ARA [29]. Hence, a low ARA/DHA ratio in membrane phospholipids, which was observed in sexually-mature monkeys in our study, appears to be a positive indicator of general neurological health. Indeed, the brain employs distinct mechanisms governing the turnover of DHA and ARA, which functions independently of each other; and also of the respective concentrations of the two PUFAs [30]. Therefore, a unique PUFA composition in neural membranes is probably instrumental to brain homeostasis and physiological functioning, which may explain the high energy expenditure partitioned to maintaining phospholipid dynamics in the brain [1].

Remarkably, our global phospholipidome analysis revealed that the reductions of ARA-containing PE and PC in sexually-mature macaques were accompanied by concomitant accumulations of their DHA-containing counterparts. Furthermore, the levels of LPE22:6 and LPC22:6 were increased at the same time. Using *Mfsd2a*-knockout mouse model, Nyugen *et al* have previously shown that plasma DHAs in the form of LPC22:6 can transverse the blood-brain barrier to enter the brain, which serve as substrates for the biosynthesis of DHA-containing phospholipids [31]. The simultaneous increases in LPE22:6 and LPC22:6, as well as DHA-containing PEs and PCs, are therefore aligned with a possibility that the major membrane lipids (*i.e*. PE and PC) in the frontal cortex of sexually-mature macaques are actively remodeled to raise their DHA content using the plasma pool of DHA as substrates. Our correlation matrix analysis revealed that considerable co-regulation exists between phospholipids comprising DHAs and CLs, which was most intense in sexually-mature monkeys and subsequently attenuated in old age. It is therefore plausible that DHA-enriched phospholipid monolayers create a membrane topology that may favour CL remodeling, thereby facilitating the maintenance of physiological level of CLs required for proper mitochondrial functions during the early and middle phases of the macaques' life. Based on this postulation, CL remodeling is possibly circumvented in the frontal cortex of old macaques as a result of attritions in PCs and PEs comprising DHAs, which explains the reductions in CLs; as well as the abated co-regulation between DHA-containing PCs and PEs with CLs in old individuals. Interestingly, the loss of co-regulation between LPC22:6 and LPE22:6 with DHA-containing membrane phospholipids implies that the diminutions of the latter in old macaques may not be predominantly attributed to a lack of DHA-containing lysophospholipid substrates for phospholipid biosynthesis/remodeling; and other factors, such as the afflicted activities of remodeling enzymes and perturbed membrane microenvironments, may be responsible for driving the reductions in DHA-phospholipids in old age. Interestingly, such mechanistic presumption is aligned with the clinical observations that while DHA diet supplementation is effective in preventing or delaying the onset of neurodegenerative diseases, no therapeutic effect of DHA supplementation was found for the treatment of established Alzheimer's disease (AD) [32].

In synaptic membranes, DHA and cholesterol represent the major determinants of membrane molecular order [4]. Notably, DHA-containing PE was shown to demonstrate a particularly strong aversion to cholesterol in membranes [3]; and that a single DHA esterified to the *sn-2* position of PE is sufficed in triggering the expulsion of sterols from model membranes [4]. Thus, the steric incompatibility between highly chaotic PUFA-rich regions and sterol-rich regions that are relatively rigid and compact serves to promote lateral segregation of membrane lipids into PUFA-rich/sterol poor and PUFA-poor/sterol-rich regions [2]. Such mutually exclusive membrane microdomains comprising either SM-/cholesterol-rich rafts or PUFA-rich/cholesterol-poor domains could serve as competing platforms that differentially regulates specific protein functions [2]. Indeed, it has been demonstrated in lymphoid cells that enriching membrane phospholipids with DHA strongly displaces phospholipase D (PLD) towards raft domains, leading to its activation [33]. Indeed, we observed reductions in frontal cortical total PC (*p* < 0.05) (Figure 1A) with concomitant increases in major PA species (*p* < 0.05) (Figure S2A) in sexually-mature macaques, for which DHA accretions in membrane phospholipids were noted.

PE serves as the primary receptacle for DHA in the brain, with an estimated 5.7 times preferential incorporation of DHA into PEs compared to PCs in cultured cells [4]. In addition, DHAs incorporated into PEs display stronger avoidance of cholesterol-rich microdomains than their corresponding PC counterparts [3]. Accordingly, while we noted enhanced levels of both PEs and PCs containing DHAs in sexually-mature monkeys in our study, only DHA-containing PEs were appreciably decreased in old animals. In concordance with this, significant accumulations of SMs with > 20C were observed in old monkeys that exhibited diminutions in DHA-containing PEs. Furthermore, our correlation matrix analysis demonstrated negative co-regulation between PS, PA and PE species comprising DHAs with VLC-SMs in old macaques, further reinforcing the idea that DHA-enriched membrane domains and raft-microdomains may operate in a segregated and perhaps mutually exclusive manner, with the latter possibly being predominant in the neuronal membranes of old macaques.

On another note, correlation matrix analyses indicated that reductions in 20:4-LBPAs were associated with increases in VLC-SMs. Previous work has implicated LBPAs in the invagination process responsible for the formation of intraluminal vesicles of multivesicular bodies (MVBs) *via* increasing local curvature of limiting membranes [34, 35]. In addition, MVBs were previously shown to facilitate degradation of long-chain SMs by transporting these lipids towards the lysosomes [36]. Thus, the selective accretions of long-chain SMs may indicate perturbations in their degradation due to compromised formation of intraluminal vesicles in MVBs, which could be attributed to the attenuated levels of polyunsaturated LBPAs in old monkeys. The exact mechanism of how polyunsaturated LBPAs outperform their saturated counterparts in terms of facilitating the degradation of long-chain SMs remains an interesting question, but a higher degree of unsaturation in the acyl chains is conventionally associated with an enhanced tendency for negative membrane curvature and vesicle formation [37].

**Figure 8:**
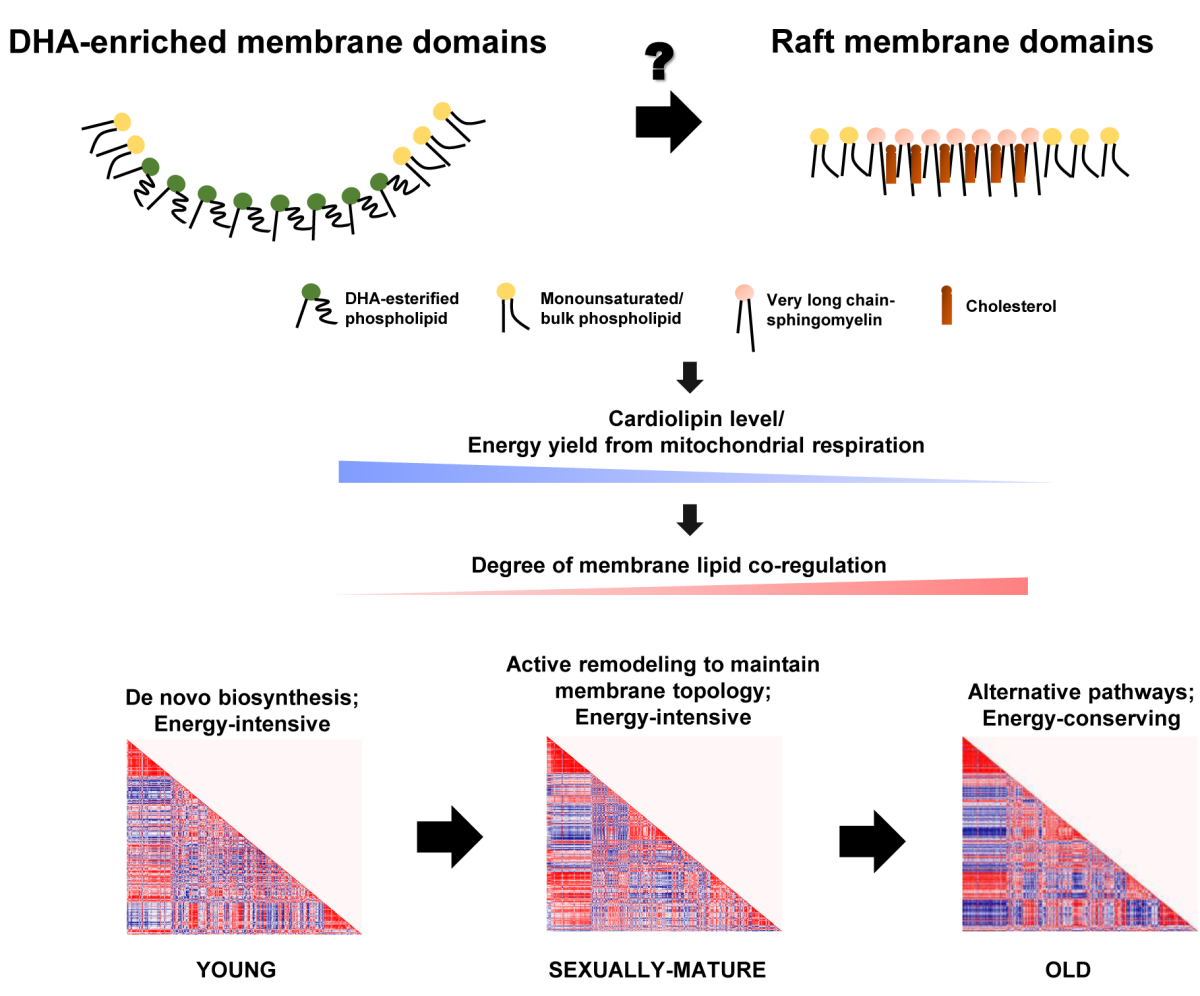
Schematic diagram illustrating the proposed role of changing membrane lipid dynamics that governs normative brain aging in *Rhesus* macaques The temporal switching of between DHA-enriched membrane microdomains and raft membrane microdomains may serve as competing platforms to switch on distinct pathways that constitute the molecular basis of aging, principally resulting in a gradual reduction in total CLs and a decline in energy availability from mitochondrial oxidative phosphorylation. The brain turns to alternative, energy-saving phospholipid synthetic pathways in place of *de novo* biosynthesis to maintain membrane dynamics under a compromised energy supply, leading to increasingly intense degree of membrane lipid co-regulation with aging.

Selective enrichment of long-chain SMs could have profound implications on membrane properties by driving the formation of ordered raft domains, of which SM represents a critical component. Our global analysis of the frontal cortical membrane lipidome across macaques of different ages therefore points to an intriguing possibility that DHA-rich microdomains and SM-/cholesterol-rich raft microdomains may operate in a temporally segregated manner, with DHA-rich microdomains dominating in sexually-mature monkeys, and a switch to a preponderance of raft-mediated regulation in old monkeys. Such temporal transitions in membrane dynamics could possibly set forth an alternate array of pathways *via* differential regulation of protein targets during the course of life, which may constitute the molecular basis of normative aging. Indeed, previous work has demonstrated that the specificity in acyl exchange reaction catalyzed by taffazin, the major CL remodeling enzyme, is conferred by the distinct membrane curvature (or the precise packing of lipids) of the mitochondrial membranes [38]. Therefore, changing membrane dynamics *per se* may account for the temporal alterations in CL profile observed in the current study, leading to a decline in energy metabolism associated with brain aging (Figure 8).

Accordingly, the drop in energy availability may herald in alternative pathways for membrane lipid synthesis that are less energy-intensive in an attempt to upkeep proper membrane dynamics in the aged brain. Notably, our correlation matrix analysis has revealed tight co-regulation between PUFA-PS and PUFA-PE that is specific to old macaques, suggestive of an enhanced level of phosphatidylserine decarboxylase (PSDC) and/or phosphatidylserine synthase 2 (PSS2) activities in the frontal cortex as normative aging progresses [39, 40]. Indeed, numerous studies employing CDP-base incorporation *in vivo* or *in vitro* have previously demonstrated that while *de novo* PC and PE biosynthesis decline appreciably with age in the brains, the content of major phospholipid classes was nevertheless maintained at an acceptable level [41–43]. These observations imply that alternative enzymatic pathways, which are probably more energy-efficient/saving than the Kennedy pathway, may set forth in aged brain tissues to maintain the physiological pools of PEs and PCs under compromised energy availability. In particular, PSS2 is known to catalyse base exchange reactions in an energy-independent but calcium-dependent manner [39], and such base exchange activities have been previously shown in the brain tissues of rats to increasingly contribute to PC and PS synthesis with aging [44]. It is noteworthy that both PSS2 and PSDC are localized to the mitochondrial membranes [39, 45]. Perturbations in membrane dynamics or membrane curvature are therefore highly likely to alter the activities of these enzymes, *via* fine-tuning their three-dimensional structures or by changing the presentation of membrane-adjacent substrates for their catalytic activities.

## CONCLUSIONS

The half-life of DHA in the brain is estimated at approximately 2.5 years [46], which is about 20 times longer than that in the rest of the body, implying that specific mechanism(s) probably exist in the brain that prioritizes its DHA requirement over other body regions in order to maintain a persistent level of DHA in neural membranes [14]. An in-depth understanding into how precisely the lipid dynamics of neural membranes may be maintained throughout the course of life could potentially unveil novel, hitherto unknown mechanisms that essentially underlie the process of normative brain aging. Indeed, clinical interventional studies have found that while an elevated habitual intake of DHA confers appreciable protection against the onset of AD, DHA supplements failed to elicit any significant improvement once AD has been diagnosed [14], suggesting that a failure in effectively incorporating/retaining DHA into neural membranes may prove more detrimental than a diminished dietary supply of DHA *per se*. It is therefore critical to understand how such mechanisms that sustain global neural membrane dynamics could fall apart during aging and neurodegeneration. In this light, Wassall and colleagues have previously postulated that the competing tendency to form either SM-/cholesterol-rich rafts or PUFA-rich/cholesterol-poor microdomains may be an avenue to fine-tune protein functions for which the individual membrane region serves as a platform [2].

Based on our global, fatty acyl-specific analysis of the frontal cortical lipidome of *Rhesus* macaques, we hypothesize that differing membrane microdomain regulation may be operative during the temporal course of normative brain aging, which initiates distinct cascades of protein functions downstream that essentially form the molecular basis of aging. In particular, the temporal switching of functional membrane microdomains may be associated with the efficiency of CL remodeling, leading to a gradual reduction in the level of total CLs and decline in energy availability from mitochondrial oxidative phosphorylation. The diminished energy supply, probably in concert with an altered membrane topology, may herald in alternative phospholipid synthetic pathways in place of *de novo* biosynthesis to maintain membrane dynamics in a more energy-saving manner, which may explain the intense degree of membrane lipid co-regulation unique to old macaques (Figure 8). Central to this idea, it appears that the key event that initiates brain aging may lie essentially in the precise mechanism that leads to a failure in maintaining a high level of esterified DHAs in neural membrane lipids.

Our study has demonstrated the sheer capacity of lipidomics to decipher the possible mechanistic basis of normative brain aging *via* capturing the fine temporal details of individual fatty acyls esterified to distinct membrane lipids during the course of aging, thereby unveiling several gerotargets and mechanistic leads central to brain aging that warrant further pursuit. The current study has several deficiencies. First, the study is circumvented by a limiting sample size, as macaque frontal cortical tissues are relatively scarce and difficult to obtain, especially for the old macaque group, which entailed keeping and rearing the monkeys for more than twenty years in enclosed facilities under standard conditions. As such, only non-parametric analyses that make no assumptions on sample size had been conducted in this study. Nevertheless, statistical results were reported prior to multiple comparison correction in the current study, as our extensive lipidomic assays coupled with a limiting sample size could have possibly eliminated several potentially meaningful gerotargets that could be further pursued *via* future mechanistic validation. Nonetheless, we have performed correction for false positives using the *q* values [47], which shown that most of the reported lipidomic changes have remained significant at *q* < 0.05 or marginally significant at *q* < 0.1 even after correction for multiple comparisons (Supplemental Tables S2-4), vindicating the validity of the proposed gerotargets and pathways in the current study.

## MATERIALS AND METHODS

### Animals and tissues

Sexual maturation occurs at 3 to 5 years of age for *Rhesus* monkeys, with an estimated median lifespan of 25 years and a maximum lifespan of 40 years [5]. Thus, the *Rhesus* macaques age at rate of approximately three times that of humans. Five male monkeys from three individual age groups were selected for the study, with a mean age of 1.5 years (young), 7.6 years (sexually-mature) and 20.4 years (old), respectively. The ages of individual monkeys were listed in Supplemental Table S1.

All monkey brain tissues were provided by the Kunming Institute of Zoology, Chinese Academy of Sciences (KIZ-CAS). All animal procedures were conducted by adhering to the international standards, and were approved in advance by the Institutional Animal Care and Use Committee of KIZ-CAS (Approval No: SYDW-2010002). Prefrontal cortex was used in the analysis, and tissues were collected immediately after the animals were euthanized and stored at −80 °C prior to use.

### Lipid extraction

The details of the extraction protocol has been previously described elsewhere [49]. Briefly, frozen tissues were inactivated by addition of 900 μL of chloroform:methanol(1:2) containing 10% deionized H_2_O. Tissues samples were homogenized on an automated bead ruptor (Omni, USA) using an optimized programme, and further incubated at 1500 rpm at 4 °C for 1 hour. At the end of the incubation, 400 μL of deionized H_2_O and 300 μL of chloroform were added to break phase. The lower organic phase was transferred to a fresh tube. A second extraction was carried out *via* addition of 500 μL of chloroform. The two extractions were pooled into the same tube and dried using SpeedVac (Genevac, UK). Samples were stored at −80 °C until mass spectrometric analysis.

### HPLC-MRM analysis

#### Mass spectrometric analysis

Quality control sample was run at the 1^st^, 8^th^ and 16^th^ sample in the queue sequence. All reported peaks were identified manually based on desirable peak shapes and signal-to-nose ratios of > 3; and demonstrated coefficients of variations of less than 12% amongst replicates of the same biological groups. Qualitative deuterated lipid standards from LIPID MAPS were pre-corrected using suitable quantitative lipid standards from the same lipid class based on molar response prior to their use for quantitation.

#### Normal-phase LC/MS

Separation of individual lipid classes of polar lipids by normal phase HPLC was carried out using a Phenomenex Luna 3u silica column (i.d. 150×2.0 mm) with the following conditions: mobile phase A (chloroform:methanol:ammonium hydroxide, 89.5:10:0.5), B (chloroform:methanol:ammonium hydroxide:water, 55:39:0.5:5.5). Multiple reaction monitoring (MRM) transitions were set up for quantitative analysis of various polar lipids. Individual lipid species were quantified by referencing to spiked internal standards. PC-14:0/14:0, PC34:1-d31, LPC-d4-26:0, PE-14:0/14:0, PE34:1-d31, LPE-17:1, PS-14:0/14:0, PS-16:0/18:1-d31, LPS-17:1, PA34:1-d31, PA-17:0/17:0, LPA-17:0, PG34:1-d31, PG-14:0/14:0, PI34:1-d31, LPI-17:1, CL-22:1(3)/14:1, LBPA-C14:0, Cer-d18:1/17:0, GluCer-d18:1/8:0, GalCer-d18:1/8:0, LacCer-d18:1/8:0, Sph-d17:0, SL-d18:1/17:0, SM-d18:1/12:0 were obtained from Avanti Polar Lipids (Alabaster, AL, USA) and LIPIDS MAPS. Dioctanoyl phosphatidylinositol (PI, 16:0-PI) was used for phosphatidylinositol quantitation and obtained from Echelon Biosciences, Inc. (Salt Lake City, UT, USA). GM3-d18:1/17:0 was synthesized in-house. For all LCMS analyses, individual peaks were manually examined and only peaks above the limit of quantitation and within the linearity range were used for quantitation. The absolute amounts of all qualitative lipid standards were pre-corrected against quantitative standards prior to their use for quantitative purposes. Details of the analytical protocol has been previously described elsewhere [49, 50].

#### Reverse-phase LC/MS

Neutral lipids (TAGs, DAGs and CEs) were analyzed using a modified version of reverse phase HPLC/ESI/MS/MS described previously [51]. Briefly, separation of lipids aforementioned was carried out on a Phenomenex Kinetex 2.6μ-C18 column (i.d. 4.6×100mm) using an isocratic mobile phase chloroform:methanol:0.1M ammonium acetate (100:100:4) at a flow rate of 150 μl/min for 22 min. Using neutral loss-based MS/MS techniques, the levels of TAG were calculated as relative contents to the spiked d5-TAG 48:0 internal standard (CDN isotopes), DAG species were quantified using 4ME 16:0 Diether DG as an internal standard (Avanti Polar Lipids, Alabaster, AL, USA) and CE species were quantified using d6-CE (CDN isotopes) as internal standard.

#### Atmospheric pressure chemical ionization (APCI)

Free cholesterols were further analyzed using HPLC/APCI/MS/MS as previously described with corresponding d6-Cho (CDN isotopes) as internal standard [52].

### Statistical analyses

Lipid levels were expressed in terms of molar fractions normalized to the total polar lipids (MFP) detected in each sample, since membrane lipid compositional changes represent the primary interest of the current study. Statistical comparisons of MFPs among young, sexually-mature and old macaques were conducted using Kruskal-Wallis non-parametric analysis of variance (ANOVA). Hierarchical clustering was performed to group lipids that displayed similar patterns of changes across the three age groups investigated, using the *pdist()* function provided in MatLab (R2012b, The MathWorks. Natick, MA, USA) with Euclidean distance metric. The details of the algorithm utilized has been previously described elsewhere [53]. Correlation matrix analysis was carried out using the non-parametric Spearman's correlation algorithm in Matlab (R2012b, The MathWorks. Natick, MA, USA). Correction for multiple comparisons were conducted using the *q* values to control for false discovery as reported previously [47]. For all analyses, **p < 0.01; *p < 0.05.

## SUPPLEMENTARY MATERIAL FIGURES AND TABLES




