# Supplementary material for: Biological relevance of fatty acyl heterogeneity to the neural membrane dynamics of Rhesus macaques during normative aging
Source: Oncotarget. 2016 Aug 10;7(35):55970–89. doi: 10.18632/oncotarget.11190 (PMC5302890; doi:10.18632/oncotarget.11190)
Supplement: Supplementary file 1 [file oncotarget-07-55970-s001.pdf]

Biological relevance of fatty acyl heterogeneity to the neural membrane dynamics of *Rhesus* macaques during normative aging

Supplementary Material

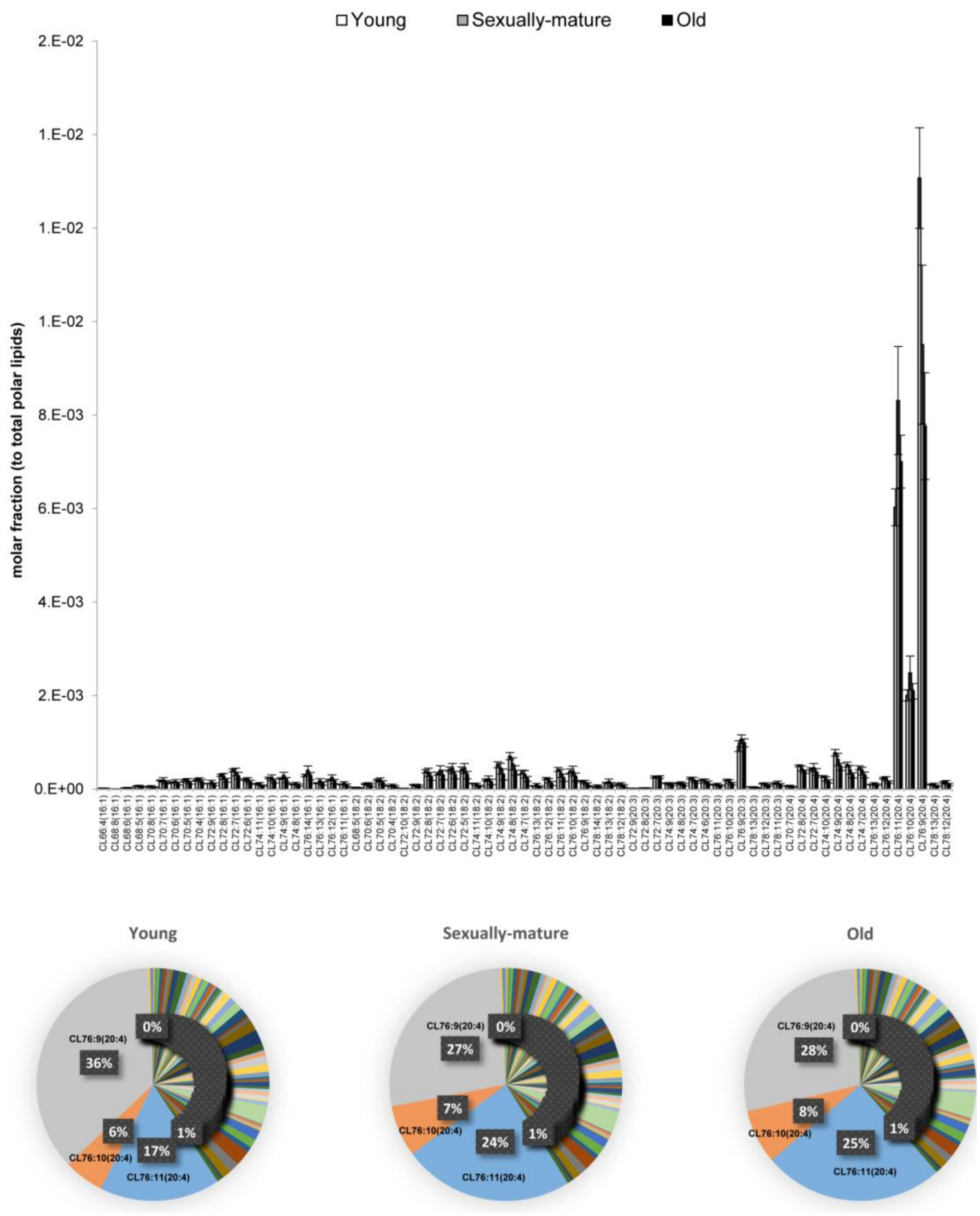

Supplemental Figure S1

Compositional profiles of all individual cardiolipins analysed. ARA-containing CLs comprise the major species in the frontal cortical CLs by abundance. Molar fractions normalized to total polar lipids were plotted. **p<0.01; *p<0.05.

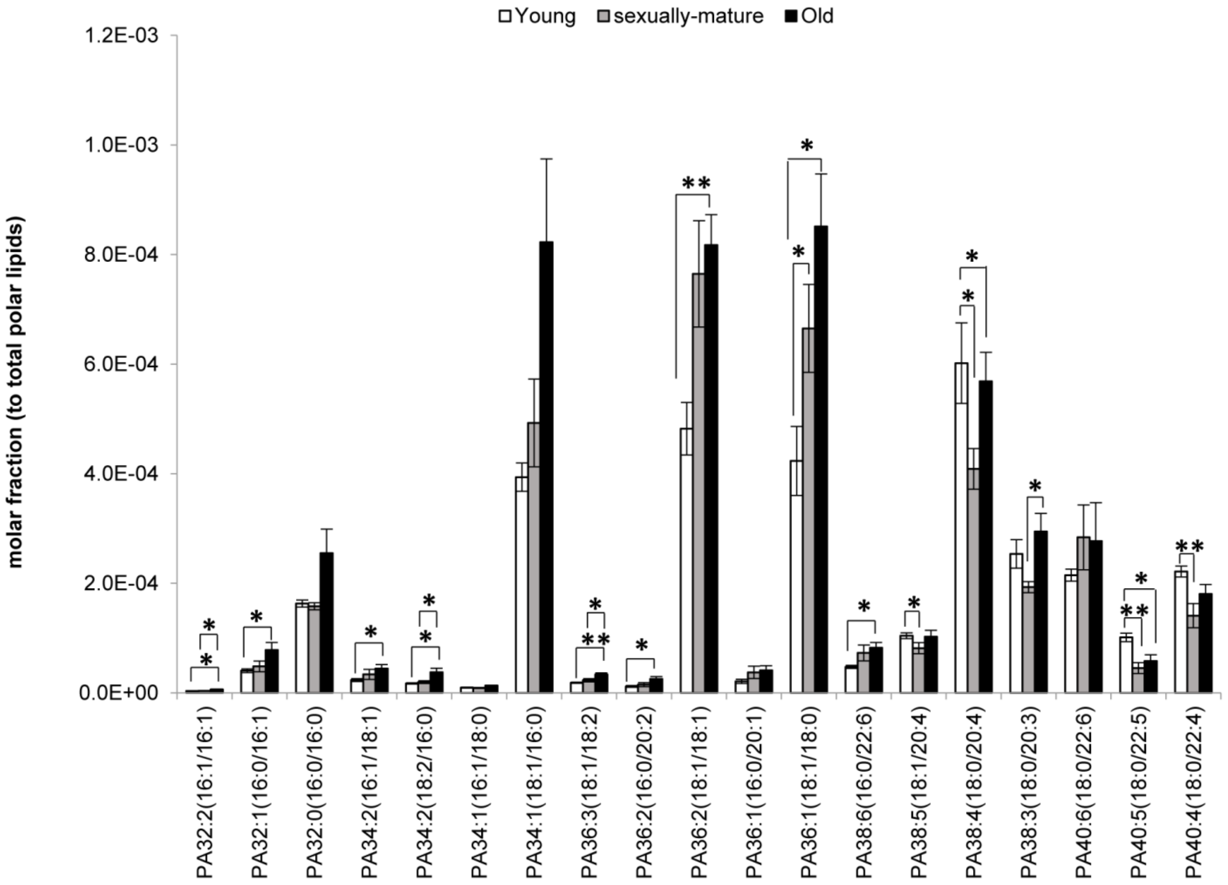

Supplemental Figure S2

Fatty acid-specific alterations in individual lysp-phosphatidylethanolamines across young, sexually-mature and old macaques. Molar fractions normalized to total polar lipids were plotted. **p<0.01; *p<0.05.

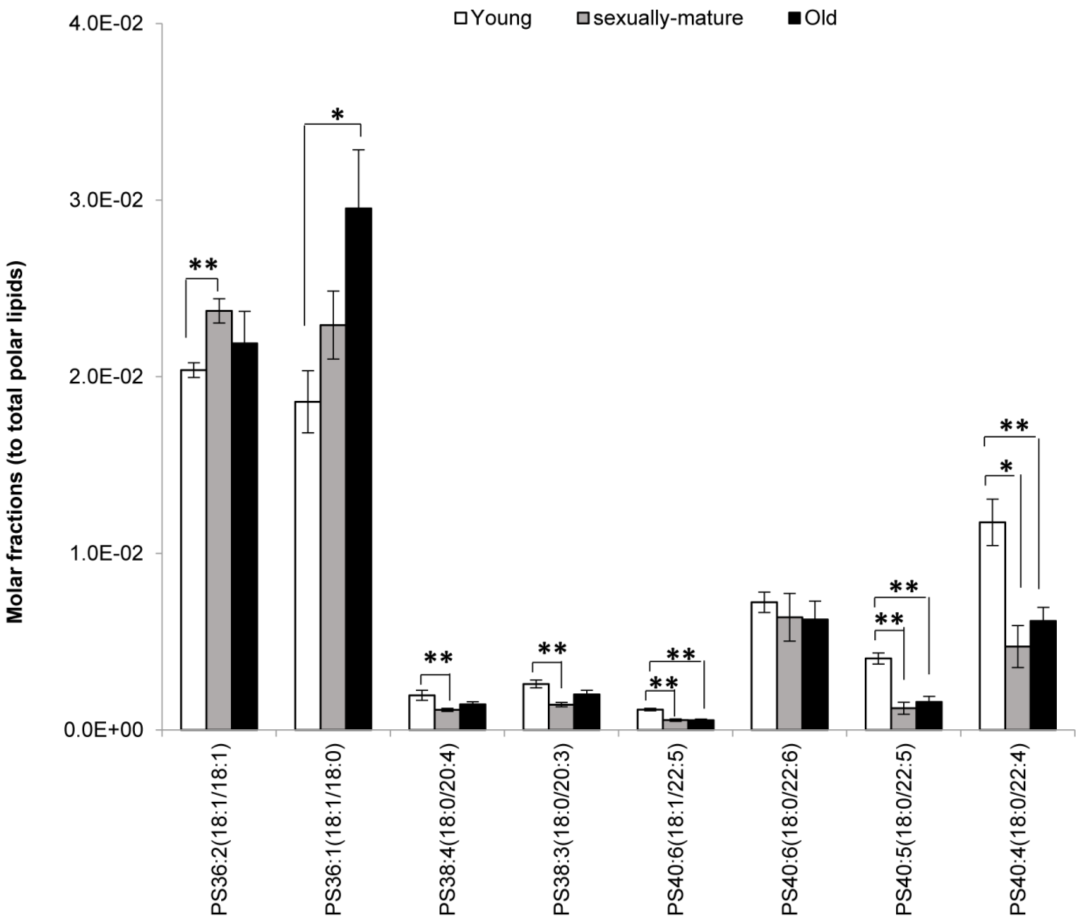

Supplemental Figure S3

Fatty acid-specific alterations in individual lyso-phosphatidylcholines across young, sexually-mature and old macaques. Molar fractions normalized to total polar lipids were plotted. **p<0.01; *p<0.05.

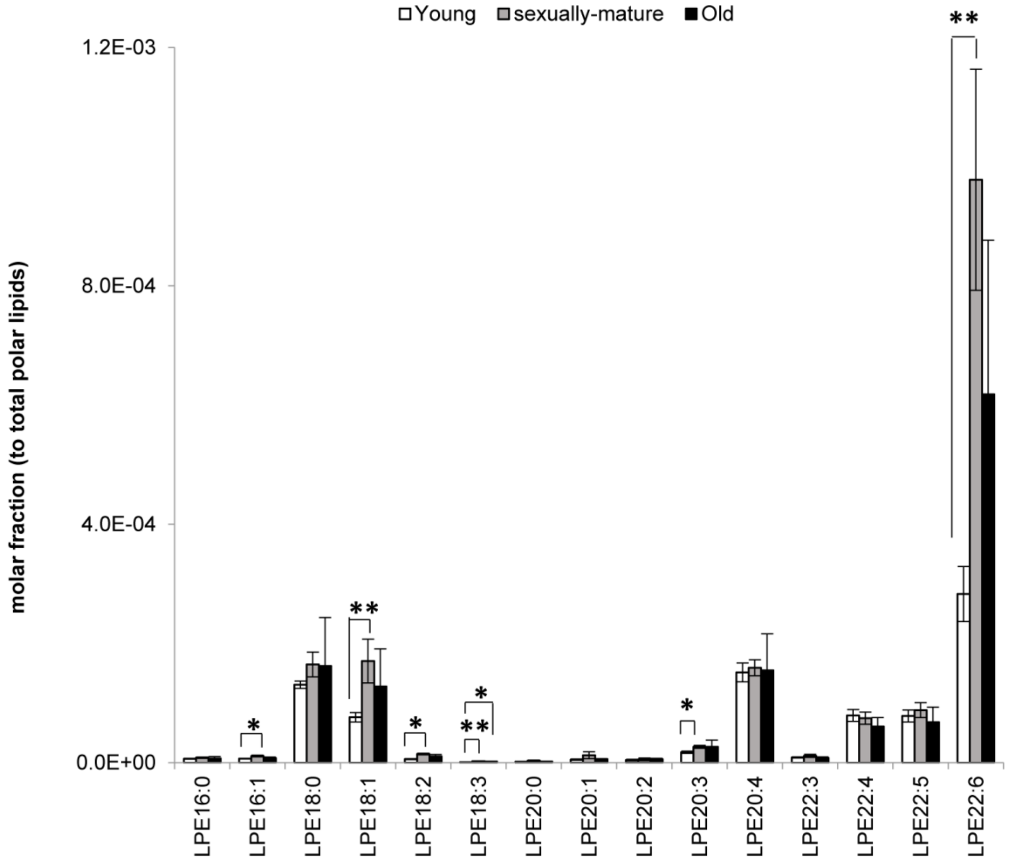

Supplemental Figure S4

Fatty acid-specific alterations in individual phosphatidic acids across young, sexually-mature and old macaques. Molar fractions normalized to total polar lipids were plotted. **p<0.01; *p<0.05.

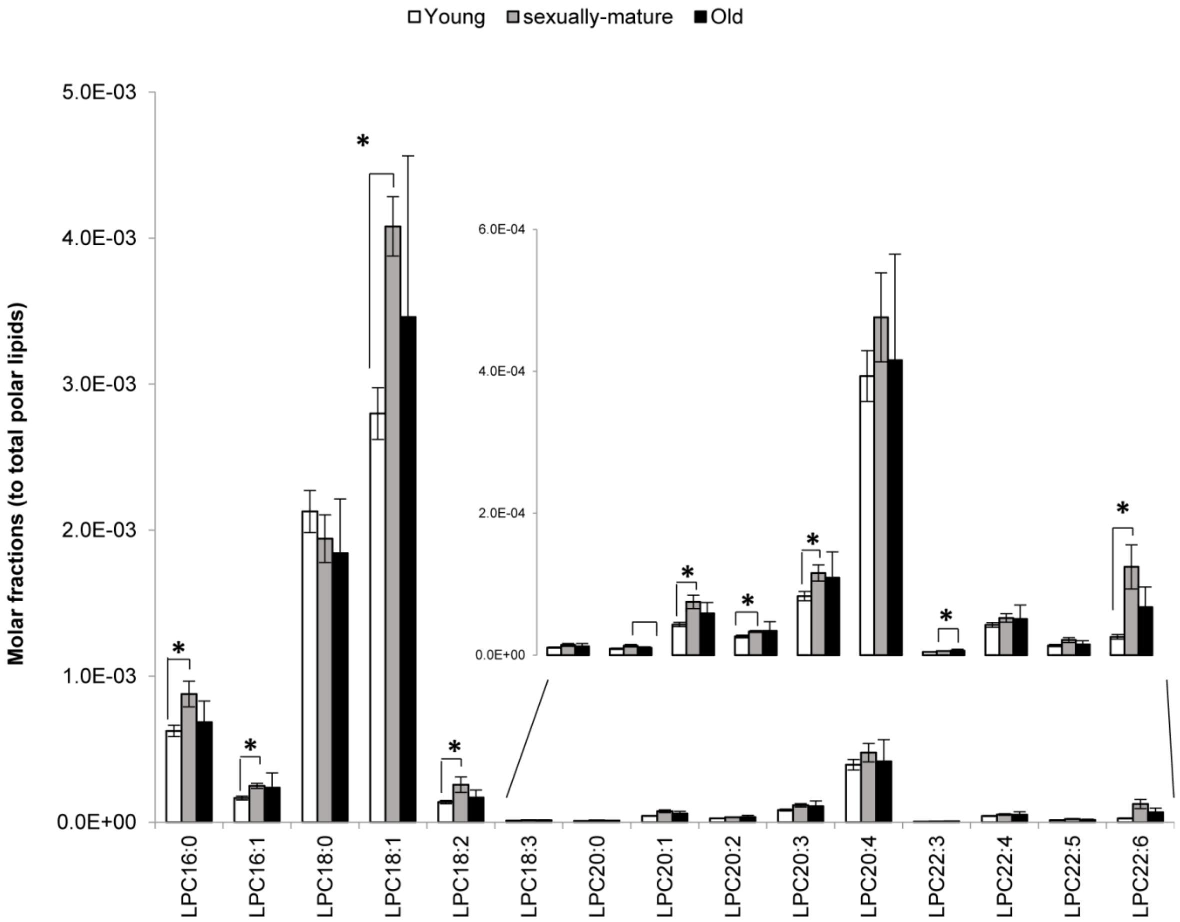

Supplemental Figure S5

Fatty acid-specific alterations in individual phosphatidylserines across young, sexually-mature and old macaques. Molar fractions normalized to total polar lipids were plotted. **p<0.01; *p<0.05.

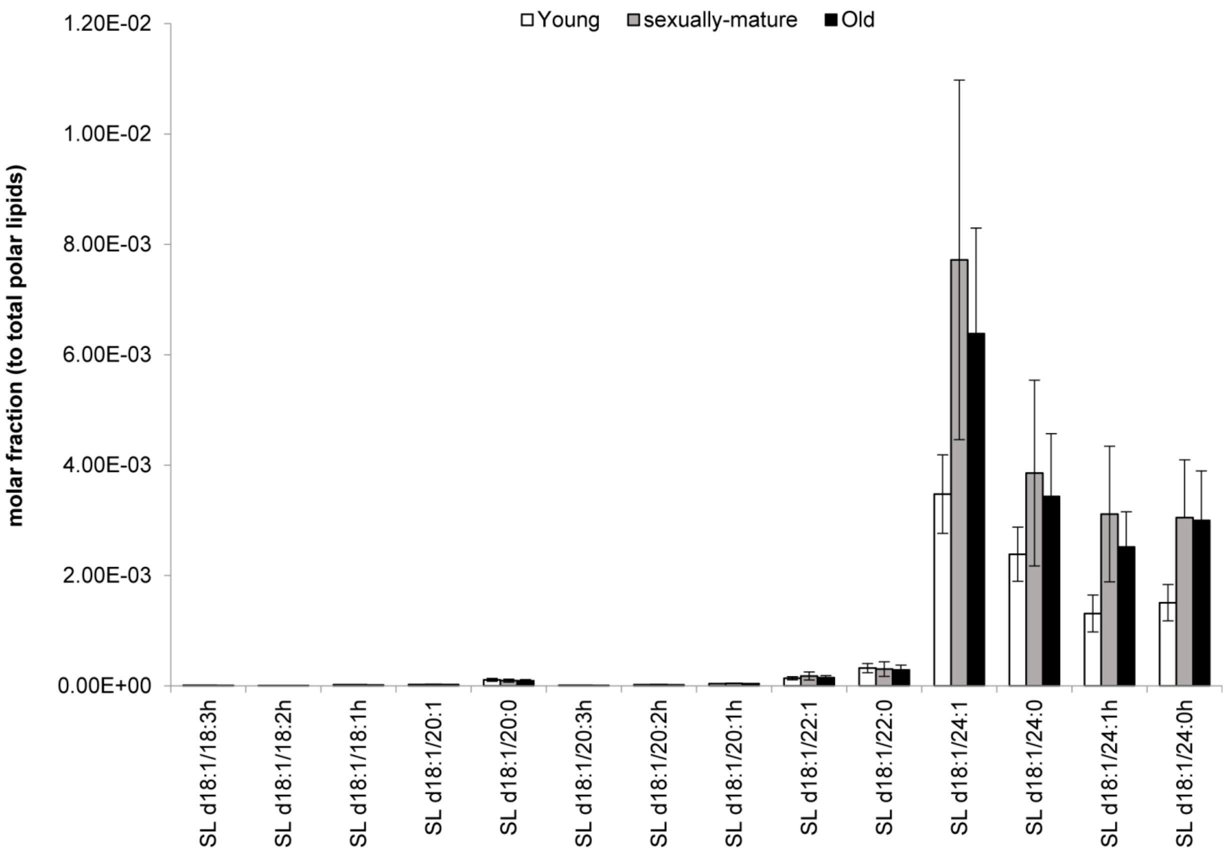

Supplemental Figure S6

Fatty acid-specific alterations in individual sulfatides across young, sexually-mature and old macaques. Molar fractions normalized to total polar lipids were plotted. **p<0.01; *p<0.05.

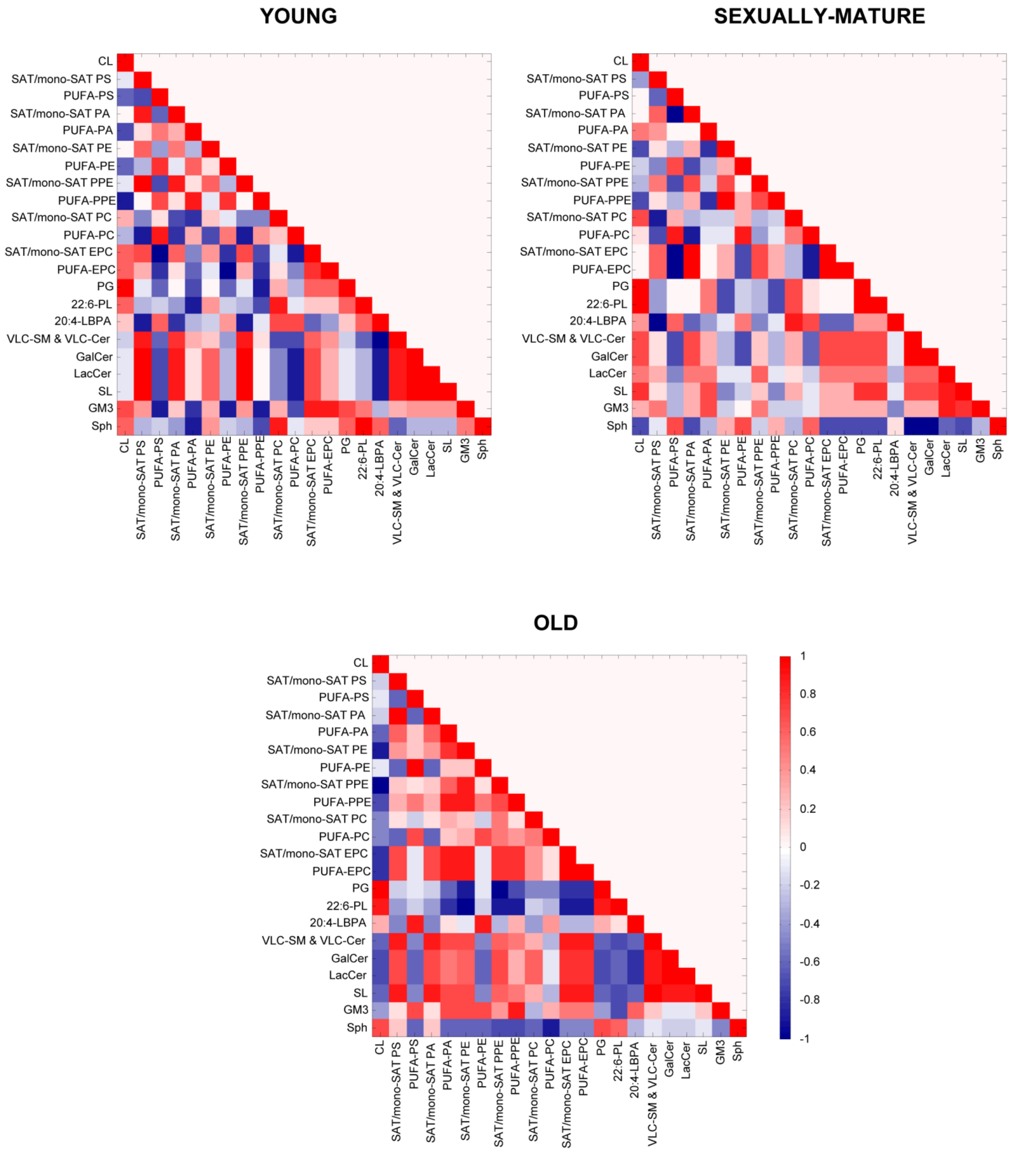

Supplemental Figure S7

Lipid correlation matrices constructed based on the membrane lipidomes of young, sexually-mature and old macaques each comprising 22 individual lipid subclasses. Vertical axis indicates magnitudes of correlation coefficients (r).
