# Supplementary material for: Biological relevance of fatty acyl heterogeneity to the neural membrane dynamics of Rhesus macaques during normative aging
Source: Oncotarget. 2016 Aug 10;7(35):55970–89. doi: 10.18632/oncotarget.11190 (PMC5302890; doi:10.18632/oncotarget.11190)
Supplement: Supplementary file 2 [file oncotarget-07-55970-s002.pdf]

Supplemental Table S1: Ages of individual monkeys investigated in the current study.

Age category	Age (years)	Region	Sex
OLD	18	PFC	M
	21	PFC	M
	20	PFC	M
	23	PFC	M
	20	PFC	M
Mean age	20.4		
SEXUALLY-MATURE	8	PFC	M
	8	PFC	M
	8	PFC	M
	6	PFC	M
	8	PFC	M
Mean age	7.6		
YOUNG	1-2	PFC	M
	1-2	PFC	M
	1-2	PFC	M
	1-2	PFC	M
	1-2	PFC	M
Mean age	1.5		

PFC: prefrontal cortex; M: male

Supplemental Table S2: P-values from Kruskal-Wallis tests and q-values after correction for false discovery for lipid comparisons listed in Figure 3.

	Mature vs Young	Mature vs Young	Old vs Young	Old vs Young
	p value	q value	p value	q value
CL70:7(20:4)	0.35	0.33	0.12	0.31
CL72:7(20:4)	0.35	0.33	0.46	0.65
CL72:8(20:4)	0.75	0.53	0.02	0.12
CL74:10(20:4)	0.46	0.39	0.08	0.25
CL74:7(20:4)	0.92	0.60	0.08	0.25
CL74:8(20:4)	0.46	0.39	0.02	0.12
CL74:9(20:4)	0.46	0.39	0.02	0.12
CL76:10(20:4)	0.46	0.39	0.60	0.72
CL76:12(20:4)	0.92	0.60	0.12	0.31
CL76:13(20:4)	0.35	0.33	0.46	0.65
CL76:9(20:4)	0.12	0.19	0.02	0.12
CL78:12(20:4)	0.75	0.53	0.25	0.47
CL78:13(20:4)	0.92	0.60	0.17	0.38
PG36:3(20:3/16:0)	0.03	0.09	0.03	0.15
PG36:4(20:3/16:1)	0.01	0.05	0.03	0.15
PG36:4(20:4/16:0)	0.01	0.05	0.01	0.09
PG38:3(20:3/18:0)	0.02	0.06	0.02	0.12
PG38:4(16:0/22:4)	0.08	0.17	0.02	0.12
PG38:4(20:4/18:0)	0.01	0.05	0.01	0.09
PG38:5(20:4/18:1)	0.01	0.05	0.01	0.09
PG38:5(20:5/18:0)	0.75	0.53	0.03	0.15
PG38:5(22:5/16:0)	0.01	0.05	0.01	0.09
PG38:6(22:6/16:0)	0.12	0.19	0.75	0.79
PE38:4p(20:4/18:0p)	0.02	0.06	0.01	0.09
PE38:5p(22:5/16:0p)	0.01	0.05	0.01	0.09
PE40:4p(22:4/18:0p)	0.01	0.05	0.01	0.09
PE40:5p(22:5/18:0p)	0.01	0.05	0.01	0.09
PE40:6p(22:5/18:1p)	0.02	0.06	0.01	0.09
PE40:6p(22:6/18:0p)	0.25	0.28	0.05	0.20
PE32:0(16:0/16:0)	0.01	0.05	0.01	0.09
PE34:0(16:0/18:0)	0.01	0.05	0.01	0.09
PE34:1(16:1/18:0)	0.03	0.09	0.01	0.09
PE34:1(18:1/16:0)	0.08	0.17	0.01	0.09
PE34:2(16:0/18:2)	0.02	0.06	0.01	0.09
PE36:1(18:1/18:0)	0.01	0.05	0.01	0.09
PE38:3(20:3/18:0)	0.01	0.05	0.01	0.09
PE38:4(16:0/22:4)	0.01	0.05	0.01	0.09
PE38:4(20:4/18:0)	0.01	0.05	0.01	0.09
PE38:5(16:0/22:5)	0.01	0.05	0.01	0.09
PE38:5(20:4/18:1)	0.02	0.06	0.01	0.09
PE38:6(22:6/16:0)	0.08	0.17	0.12	0.31
PE38:7(22:6/16:1)	0.60	0.46	0.02	0.12
PE40:4(18:0/22:4)	0.01	0.05	0.01	0.09
PE40:5(22:5/18:0)	0.01	0.05	0.01	0.09
PE40:6(22:6/18:0)	0.25	0.28	0.12	0.31
PC30:0(16:0/14:0)	0.02	0.06	0.05	0.20
PC32:0(16:0/16:0)	0.46	0.39	0.60	0.72
PC32:1(16:1/16:0)	0.08	0.17	0.46	0.65
PC34:0(16:0/18:0)	0.17	0.23	0.17	0.38
PC34:1(16:1/18:0)	0.08	0.17	0.46	0.65
PC34:2(16:1/18:1)	0.01	0.05	0.25	0.47
PC34:2(18:1/16:1)	0.60	0.46	0.35	0.57
PC36:1(18:1/18:0)	0.17	0.23	0.92	0.87
PC36:2(18:1/18:1)	0.25	0.28	0.60	0.72
PC36:3(20:3/16:0)	0.05	0.13	0.92	0.87
PC36:4(16:0/20:4)	0.03	0.09	0.03	0.15
PC36:4(20:4/16:0)	0.03	0.09	0.03	0.15
PC38:3(20:3/18:0)	0.01	0.05	0.46	0.65
PC38:4(20:3/18:1)	0.01	0.05	0.92	0.87
PC38:4(20:4/18:0)	0.01	0.05	0.03	0.15
PC38:4(22:4/16:0)	0.01	0.05	0.35	0.57
PC38:5(20:4/18:1)	0.01	0.05	0.03	0.15
PC38:6(20:4/18:2)	0.05	0.13	0.03	0.15
PC38:6(22:5/16:1)	0.03	0.09	0.05	0.20
PC38:6(22:6/16:0)	0.01	0.05	0.12	0.31
PC40:6(22:6/18:0)	0.01	0.05	0.25	0.47
PC40:7(22:6/18:1)	0.01	0.05	0.35	0.57

Supplemental Table S3: P-values from Kruskal-Wallis tests and q-values after correction for false discovery for lipid comparisons listed in Figure 4.

	Mature vs Young	Mature vs Young	Old vs Young	Old vs Young
	p value	q value	p value	q value
Cer d18:0/16:0	0.46	0.39	0.75	0.79
Cer d18:0/18:0	0.01	0.05	0.12	0.31
Cer d18:0/20:0	0.35	0.33	0.60	0.72
Cer d18:0/22:0	0.02	0.06	0.75	0.79
Cer d18:0/24:0	0.75	0.53	0.25	0.47
Cer d18:0/24:1	0.05	0.13	0.60	0.72
Cer d18:1/16:0	0.25	0.28	0.17	0.38
Cer d18:1/18:0	0.01	0.05	0.12	0.31
Cer d18:1/20:0	0.92	0.60	0.35	0.57
Cer d18:1/22:0	0.02	0.06	0.46	0.65
Cer d18:1/24:0	0.02	0.06	0.75	0.79
Cer d18:1/24:1	0.05	0.13	0.60	0.72
GalCer d18:0/16:0	0.46	0.39	0.08	0.25
GalCer d18:0/18:0	0.17	0.23	0.92	0.87
GalCer d18:0/20:0	0.17	0.23	0.75	0.79
GalCer d18:0/22:0	0.12	0.19	0.35	0.57
GalCer d18:0/24:0	0.12	0.19	0.46	0.65
GalCer d18:0/24:1	0.12	0.19	0.75	0.79
GalCer d18:1/16:0	0.12	0.19	0.60	0.72
GalCer d18:1/18:0	0.17	0.23	0.75	0.79
GalCer d18:1/20:0	0.46	0.39	0.35	0.57
GalCer d18:1/22:0	0.12	0.19	0.75	0.79
GalCer d18:1/24:0	0.17	0.23	0.75	0.79
GalCer d18:1/24:1	0.17	0.23	0.92	0.87
LacCer d18:0/16:0	0.12	0.19	0.17	0.38
LacCer d18:0/18:0	0.05	0.13	0.12	0.31
LacCer d18:0/20:0	0.35	0.33	0.35	0.57
LacCer d18:0/22:0	0.12	0.19	0.12	0.31
LacCer d18:0/24:0	0.03	0.09	0.03	0.15
LacCer d18:0/24:1	0.05	0.13	0.03	0.15
LacCer d18:1/16:0	0.46	0.39	0.60	0.72
LacCer d18:1/18:0	0.03	0.09	0.08	0.25
LacCer d18:1/20:0	0.08	0.17	0.08	0.25
LacCer d18:1/22:0	0.60	0.46	0.12	0.31
LacCer d18:1/24:0	0.05	0.13	0.03	0.15
LacCer d18:1/24:1	0.05	0.13	0.02	0.12
GM3 d18:0/16:0	0.01	0.05	0.03	0.15
GM3 d18:0/18:0	0.01	0.05	0.03	0.15
GM3 d18:0/20:0	0.02	0.06	0.25	0.47
GM3 d18:0/22:0	0.01	0.05	0.01	0.09
GM3 d18:1/16:0	0.12	0.19	0.60	0.72
GM3 d18:1/18:0	0.01	0.05	0.02	0.12
GM3 d18:1/18:1	0.03	0.09	0.12	0.31
GM3 d18:1/20:0	0.03	0.09	0.46	0.65
GM3 d18:1/20:1	0.01	0.05	0.46	0.65
GM3 d18:1/22:0	0.01	0.05	0.03	0.15
GM3 d18:1/22:1	0.02	0.06	0.25	0.47
SM d18:0/16:0	0.17	0.23	0.01	0.09
SM d18:0/18:0	0.05	0.13	0.60	0.72
SM d18:0/24:0	0.12	0.19	0.08	0.25
SM d18:1/16:0	0.75	0.53	0.12	0.31
SM d18:1/16:1	0.17	0.23	0.12	0.31
SM d18:1/18:0	0.03	0.09	0.60	0.72
SM d18:1/18:1	0.17	0.23	0.75	0.79
SM d18:1/20:0	0.01	0.05	0.05	0.20
SM d18:1/20:1	0.60	0.46	0.35	0.57
SM d18:1/22:0	0.46	0.39	0.01	0.09
SM d18:1/22:1	0.35	0.33	0.17	0.38
SM d18:1/24:0	0.08	0.17	0.03	0.15
SM d18:1/24:1	0.25	0.28	0.03	0.15
LBPA32:1(16:0/16:1)	0.17	0.23	0.92	0.87
LBPA32:2(16:1/16:1)	0.92	0.60	0.92	0.87
LBPA34:1(16:1/18:0)	0.92	0.60	0.92	0.87
LBPA34:1(18:1/16:0)	0.75	0.53	0.08	0.25
LBPA34:2(18:1/16:1)	0.08	0.17	0.46	0.65
LBPA34:2(18:2/16:0)	0.35	0.33	0.35	0.57
LBPA34:3(18:2/16:1)	0.46	0.39	0.25	0.47
LBPA36:1(16:0/20:1)	0.46	0.39	0.08	0.25
LBPA36:1(18:1/18:0)	0.35	0.33	0.60	0.72
LBPA36:2(18:1/18:1)	0.12	0.19	0.08	0.25
LBPA36:2(18:2/18:0)	0.75	0.53	0.92	0.87
LBPA36:3(18:2/18:1)	0.92	0.60	0.25	0.47
LBPA36:3(20:3/16:0)	0.05	0.13	0.35	0.57
LBPA36:4(18:2/18:2)	0.46	0.39	0.46	0.65
LBPA36:4(20:3/16:1)	0.17	0.23	0.25	0.47
LBPA36:4(20:4/16:0)	0.01	0.05	0.01	0.09
LBPA38:3(16:0/22:3)	0.75	0.53	0.35	0.57
LBPA38:3(20:3/18:0)	0.01	0.05	0.05	0.20
LBPA38:4(16:0/22:4)	0.08	0.17	0.01	0.09
LBPA38:4(20:3/18:1)	0.92	0.60	0.08	0.25
LBPA38:4(20:4/18:0)	0.01	0.05	0.01	0.09
LBPA38:5(20:4/18:1)	0.01	0.05	0.01	0.09
LBPA38:5(20:5/18:0)	0.92	0.60	0.60	0.72
LBPA38:5(22:5/16:0)	0.25	0.28	0.12	0.31
LBPA38:6(20:5/18:1)	0.12	0.19	0.17	0.38
LBPA38:6(22:6/16:0)	0.46	0.39	0.46	0.65

Supplemental Table S4: P-values from Kruskal-Wallis tests and q-values after correction for false discovery for lipid comparisons listed in Supplemental Figures S2-5.

	Mature vs Young	Mature vs Young	Old vs Young	Old vs Young
	P value	q value	P value	q value
PA32:0(16:0/16:0)	0.60	0.46	0.12	0.31
PA32:1(16:0/16:1)	0.75	0.53	0.03	0.15
PA32:2(16:1/16:1)	0.92	0.60	0.03	0.15
PA34:1(16:1/18:0)	0.60	0.46	0.05	0.20
PA34:1(18:1/16:0)	0.17	0.23	0.08	0.25
PA34:2(16:1/18:1)	0.35	0.33	0.05	0.20
PA34:2(18:2/16:0)	0.75	0.53	0.08	0.25
PA36:1(18:1/18:0)	0.05	0.13	0.02	0.12
PA36:2(16:0/20:2)	0.75	0.53	0.03	0.15
PA36:2(18:1/18:1)	0.08	0.17	0.01	0.09
PA36:3(18:1/18:2)	0.35	0.33	0.01	0.09
PA38:3(18:0/20:3)	0.12	0.19	0.60	0.72
PA38:4(18:0/20:4)	0.03	0.09	0.92	0.87
PA38:5(18:1/20:4)	0.03	0.09	0.92	0.87
PA38:6(16:0/22:6)	0.17	0.23	0.02	0.12
PA40:4(22:4/18:0)	0.01	0.05	0.08	0.25
PA40:5(22:5/18:0)	0.01	0.05	0.03	0.15
PS36:1(18:1/18:0)	0.17	0.23	0.05	0.20
PS36:2(18:1/18:1)	0.01	0.05	0.60	0.72
PS38:3(20:3/18:0)	0.01	0.05	0.08	0.25
PS38:4(20:4/18:0)	0.01	0.05	0.12	0.31
PS40:4(22:4/18:0)	0.02	0.06	0.01	0.09
PS40:5(22:5/18:0)	0.01	0.05	0.01	0.09
PS40:6(18:1/22:5)	0.01	0.05	0.01	0.09
PS40:6(22:6/18:0)	0.75	0.53	0.46	0.65
LPE16:0	0.17	0.23	0.17	0.38
LPE16:1	0.02	0.06	0.92	0.87
LPE18:0	0.17	0.23	0.12	0.31
LPE18:1	0.01	0.05	0.75	0.79
LPE18:2	0.01	0.05	0.46	0.65
LPE18:3	0.01	0.05	0.05	0.20
LPE20:0	0.25	0.28	0.92	0.87
LPE20:1	0.17	0.23	0.60	0.72
LPE20:2	0.92	0.60	0.92	0.87
LPE20:3	0.03	0.09	0.75	0.79
LPE20:4	0.60	0.46	0.35	0.57
LPE22:3	0.92	0.60	0.35	0.57
LPE22:4	0.60	0.46	0.35	0.57
LPE22:5	0.60	0.46	0.25	0.47
LPE22:6	0.01	0.05	0.60	0.72
LPC16:0	0.05	0.13	0.75	0.79
LPC16:1	0.02	0.06	0.46	0.65
LPC18:0	0.35	0.33	0.12	0.31
LPC18:1	0.02	0.06	0.60	0.72
LPC18:2	0.05	0.13	0.75	0.79
LPC18:3	0.12	0.19	0.75	0.79
LPC20:0	0.05	0.13	0.46	0.65
LPC20:1	0.01	0.05	0.92	0.87
LPC20:2	0.03	0.09	0.60	0.72
LPC20:3	0.03	0.09	0.75	0.79
LPC20:4	0.35	0.33	0.46	0.65
LPC22:3	0.05	0.13	0.75	0.79
LPC22:4	0.12	0.19	0.25	0.47
LPC22:5	0.12	0.19	0.75	0.79
LPC22:6	0.01	0.05	0.60	0.72

Supplemental Table S5: P-values from correlation matrix constructed based on 22 individual lipid subclasses in young macaques.

	CL	SAT/mono-SAT PS	PUFA-PS	SAT/mono-SAT PA	PUFA-PA	SAT/mono-SAT PE	PUFA-PE	SAT/mono-SAT PPE	PUFA-PPE	SAT/mono-SAT PC	PUFA-PC	SAT/mono-SAT EPC	PUFA-EPC	PG	22:6-PL	20:4-LBPA	VLC-SM & VLC-Cer	GalCer	LacCer	SL	GM3	Sph
CL	1.00	0.95	0.35	1.00	0.23	1.00	0.35	0.95	0.08	0.68	0.68	0.35	0.35	0.02	0.35	0.78	0.78	0.95	0.95	0.95	0.23	0.35
SAT/mono-SAT PS	0.95	1.00	0.23	0.08	0.95	0.35	0.68	0.02	1.00	0.45	0.08	0.23	0.68	0.95	0.68	0.08	0.08	0.02	0.02	0.02	0.52	0.68
PUFA-PS	0.35	0.23	1.00	0.35	0.45	0.52	0.13	0.23	0.23	0.95	0.08	0.02	0.13	0.35	0.78	0.35	0.35	0.23	0.23	0.23	0.08	0.78
SAT/mono-SAT PA	1.00	0.08	0.35	1.00	0.68	0.45	0.95	0.08	0.95	0.23	0.13	0.35	0.95	1.00	0.52	0.13	0.13	0.08	0.08	0.08	0.78	0.52
PUFA-PA	0.23	0.95	0.45	0.68	1.00	0.68	0.35	0.95	0.08	0.13	0.68	0.45	0.35	0.23	0.08	0.68	0.68	0.95	0.95	0.95	0.13	0.08
SAT/mono-SAT PE	1.00	0.35	0.52	0.45	0.68	1.00	0.95	0.35	0.95	0.78	0.23	0.52	0.95	1.00	0.52	0.78	0.78	0.35	0.35	0.35	0.68	0.52
PUFA-PE	0.35	0.68	0.13	0.95	0.35	0.95	1.00	0.68	0.13	0.95	0.45	0.13	0.02	0.35	0.78	0.52	0.52	0.68	0.68	0.68	0.08	0.78
SAT/mono-SAT PPE	0.95	0.02	0.23	0.08	0.95	0.35	0.68	1.00	1.00	0.45	0.08	0.23	0.68	0.95	0.68	0.08	0.08	0.02	0.02	0.02	0.52	0.68
PUFA-PPE	0.08	1.00	0.23	0.95	0.08	0.95	0.13	1.00	1.00	0.45	0.52	0.23	0.13	0.08	0.23	0.95	0.95	1.00	1.00	1.00	0.08	0.23
SAT/mono-SAT PC	0.68	0.45	0.95	0.23	0.13	0.78	0.95	0.45	0.45	1.00	0.78	0.95	0.95	0.68	0.08	0.23	0.23	0.45	0.45	0.45	0.68	0.08
PUFA-PC	0.68	0.08	0.08	0.13	0.68	0.23	0.45	0.08	0.52	0.78	1.00	0.08	0.45	0.68	0.95	0.23	0.23	0.08	0.08	0.08	0.23	0.95
SAT/mono-SAT EPC	0.35	0.23	0.02	0.35	0.45	0.52	0.13	0.23	0.23	0.95	0.08	1.00	0.13	0.35	0.78	0.35	0.35	0.23	0.23	0.23	0.08	0.78
PUFA-EPC	0.35	0.68	0.13	0.95	0.35	0.95	0.02	0.68	0.13	0.95	0.45	0.13	1.00	0.35	0.78	0.52	0.52	0.68	0.68	0.68	0.08	0.78
PG	0.02	0.95	0.35	1.00	0.23	1.00	0.35	0.95	0.08	0.68	0.68	0.35	0.35	1.00	0.35	0.78	0.78	0.95	0.95	0.95	0.23	0.35
22:6-PL	0.35	0.68	0.78	0.52	0.08	0.52	0.78	0.68	0.23	0.08	0.95	0.78	0.78	0.35	1.00	0.35	0.35	0.68	0.68	0.68	0.45	0.02
20:4-LBPA	0.78	0.08	0.35	0.13	0.68	0.78	0.52	0.08	0.95	0.23	0.23	0.35	0.52	0.78	0.35	1.00	0.02	0.08	0.08	0.08	0.68	0.35
VLC-SM & VLC-Cer	0.78	0.08	0.35	0.13	0.68	0.78	0.52	0.08	0.95	0.23	0.23	0.35	0.52	0.78	0.35	0.02	1.00	0.08	0.08	0.08	0.68	0.35
GalCer	0.95	0.02	0.23	0.08	0.95	0.35	0.68	0.02	1.00	0.45	0.08	0.23	0.68	0.95	0.68	0.08	0.08	1.00	0.02	0.02	0.52	0.68
LacCer	0.95	0.02	0.23	0.08	0.95	0.35	0.68	0.02	1.00	0.45	0.08	0.23	0.68	0.95	0.68	0.08	0.08	0.02	1.00	0.02	0.52	0.68
SL	0.95	0.02	0.23	0.08	0.95	0.35	0.68	0.02	1.00	0.45	0.08	0.23	0.68	0.95	0.68	0.08	0.08	0.02	0.02	1.00	0.52	0.68
GM3	0.23	0.52	0.08	0.78	0.13	0.68	0.08	0.52	0.08	0.68	0.23	0.08	0.08	0.23	0.45	0.68	0.68	0.52	0.52	0.52	1.00	0.45
Sph	0.35	0.68	0.78	0.52	0.08	0.52	0.78	0.68	0.23	0.08	0.95	0.78	0.78	0.35	0.02	0.35	0.35	0.68	0.68	0.68	0.45	1.00

Supplemental Table S6: P-values from correlation matrix constructed based on 22 individual lipid subclasses in sexually-mature macaques.

	CL	SAT/mono-SAT PS	PUFA-PS	SAT/mono-SAT PA	PUFA-PA	SAT/mono-SAT PE	PUFA-PE	SAT/mono-SAT PPE	PUFA-PPE	SAT/mono-SAT PC	PUFA-PC	SAT/mono-SAT EPC	PUFA-EPC	PG	22:6-PL	20:4-LBPA	VLC-SM & VLC-Cer	GalCer	LacCer	SL	GM3	Sph
CL	1.00	0.52	1.00	1.00	0.45	0.23	0.78	0.68	0.23	0.23	0.95	1.00	1.00	0.02	0.02	0.52	0.23	0.23	0.45	0.13	0.68	0.23
SAT/mono-SAT PS	0.52	1.00	0.35	0.35	0.52	0.95	0.45	0.45	0.95	0.08	0.23	0.35	0.35	0.52	0.52	0.02	0.95	0.95	0.68	0.95	0.45	0.95
PUFA-PS	1.00	0.35	1.00	0.02	1.00	0.68	0.23	0.23	0.68	0.68	0.08	0.02	0.02	1.00	1.00	0.35	0.23	0.23	0.45	0.68	0.68	0.23
SAT/mono-SAT PA	1.00	0.35	0.02	1.00	1.00	0.68	0.23	0.23	0.68	0.68	0.08	0.02	0.02	1.00	1.00	0.35	0.23	0.23	0.45	0.68	0.68	0.23
PUFA-PA	0.45	0.52	1.00	1.00	1.00	0.13	0.68	0.78	0.13	0.78	0.95	1.00	1.00	0.45	0.45	0.52	0.68	0.68	0.45	0.23	0.23	0.68
SAT/mono-SAT PE	0.23	0.95	0.68	0.68	0.13	1.00	0.68	0.23	0.02	0.78	0.95	0.68	0.68	0.23	0.23	0.95	0.68	0.68	0.95	0.45	0.78	0.68
PUFA-PE	0.78	0.45	0.23	0.23	0.68	0.68	1.00	1.00	0.68	0.68	0.08	0.23	0.23	0.78	0.78	0.45	0.23	0.23	0.95	0.78	1.00	0.23
SAT/mono-SAT PPE	0.68	0.45	0.23	0.23	0.78	0.23	1.00	1.00	0.23	0.68	0.52	0.23	0.23	0.68	0.68	0.45	0.78	0.78	0.35	0.78	0.45	0.78
PUFA-PPE	0.23	0.95	0.68	0.68	0.13	0.02	0.68	0.23	1.00	0.78	0.95	0.68	0.68	0.23	0.23	0.95	0.68	0.68	0.95	0.45	0.78	0.68
SAT/mono-SAT PC	0.23	0.08	0.68	0.68	0.78	0.78	0.68	0.68	0.78	1.00	0.45	0.68	0.68	0.23	0.23	0.08	0.68	0.68	0.95	0.68	0.78	0.68
PUFA-PC	0.95	0.23	0.08	0.08	0.95	0.95	0.08	0.52	0.95	0.45	1.00	0.08	0.08	0.95	0.95	0.23	0.35	0.35	0.78	0.95	0.95	0.35
SAT/mono-SAT EPC	1.00	0.35	0.02	0.02	1.00	0.68	0.23	0.23	0.68	0.68	0.08	1.00	0.02	1.00	1.00	0.35	0.23	0.23	0.45	0.68	0.68	0.23
PUFA-EPC	1.00	0.35	0.02	0.02	1.00	0.68	0.23	0.23	0.68	0.68	0.08	0.02	1.00	1.00	1.00	0.35	0.23	0.23	0.45	0.68	0.68	0.23
PG	0.02	0.52	1.00	1.00	0.45	0.23	0.78	0.68	0.23	0.23	0.95	1.00	1.00	1.00	0.02	0.52	0.23	0.23	0.45	0.13	0.68	0.23
22:6-PL	0.02	0.52	1.00	1.00	0.45	0.23	0.78	0.68	0.23	0.23	0.95	1.00	1.00	0.02	1.00	0.52	0.23	0.23	0.45	0.13	0.68	0.23
20:4-LBPA	0.52	0.02	0.35	0.35	0.52	0.95	0.45	0.45	0.95	0.08	0.23	0.35	0.35	0.52	0.52	1.00	0.95	0.95	0.68	0.95	0.45	0.95
VLC-SM & VLC-Cer	0.23	0.95	0.23	0.23	0.68	0.68	0.23	0.78	0.68	0.68	0.35	0.23	0.23	0.23	0.23	0.95	1.00	0.02	0.35	0.23	0.68	0.02
GalCer	0.23	0.95	0.23	0.23	0.68	0.68	0.23	0.78	0.68	0.68	0.35	0.23	0.23	0.23	0.23	0.95	0.02	1.00	0.35	0.23	0.68	0.02
LacCer	0.45	0.68	0.45	0.45	0.45	0.95	0.95	0.35	0.95	0.95	0.78	0.45	0.45	0.45	0.45	0.68	0.35	0.35	1.00	0.08	0.08	0.35
SL	0.13	0.95	0.68	0.68	0.23	0.45	0.78	0.78	0.45	0.68	0.95	0.68	0.68	0.13	0.13	0.95	0.23	0.23	0.08	1.00	0.13	0.23
GM3	0.68	0.45	0.68	0.68	0.23	0.78	1.00	0.45	0.78	0.78	0.95	0.68	0.68	0.68	0.68	0.45	0.68	0.68	0.08	0.13	1.00	0.68
Sph	0.23	0.95	0.23	0.23	0.68	0.68	0.23	0.78	0.68	0.68	0.35	0.23	0.23	0.23	0.23	0.95	0.02	0.02	0.35	0.23	0.68	1.00

Supplemental Table S7: P-values from correlation matrix constructed based on 22 individual lipid subclasses in old macaques.

	CL	SAT/mono-SAT PS	PUFA-PS	SAT/mono-SAT PA	PUFA-PA	SAT/mono-SAT PE	PUFA-PE	SAT/mono-SAT PPE	PUFA-PPE	SAT/mono-SAT PC	PUFA-PC	SAT/mono-SAT EPC	PUFA-EPC	PG	22:6-PL	20:4-LBPA	VLC-SM & VLC-Cer	GalCer	LacCer	SL	GM3	Sph
CL	1.00	0.78	0.95	0.78	0.35	0.08	0.95	0.02	0.23	0.45	0.45	0.13	0.13	0.02	0.08	0.68	0.35	0.23	0.23	0.35	0.52	0.23
SAT/mono-SAT PS	0.78	1.00	0.35	0.02	0.35	0.52	0.35	0.78	0.68	0.95	0.35	0.23	0.23	0.78	0.52	0.45	0.08	0.23	0.23	0.08	0.95	0.78
PUFA-PS	0.95	0.35	1.00	0.35	0.78	0.78	0.02	0.95	0.45	0.78	0.23	0.95	0.95	0.95	0.78	0.08	0.45	0.35	0.35	0.45	0.23	0.35
SAT/mono-SAT PA	0.78	0.02	0.35	1.00	0.35	0.52	0.35	0.78	0.68	0.95	0.35	0.23	0.23	0.78	0.52	0.45	0.08	0.23	0.23	0.08	0.95	0.78
PUFA-PA	0.35	0.35	0.78	0.35	1.00	0.13	0.78	0.35	0.08	0.68	0.78	0.08	0.08	0.35	0.13	0.95	0.23	0.45	0.45	0.23	0.23	0.35
SAT/mono-SAT PE	0.08	0.52	0.78	0.52	0.13	1.00	0.78	0.08	0.08	0.78	0.68	0.08	0.08	0.08	0.02	0.95	0.23	0.35	0.35	0.23	0.23	0.35
PUFA-PE	0.95	0.35	0.02	0.35	0.78	0.78	1.00	0.95	0.45	0.78	0.23	0.95	0.95	0.95	0.78	0.08	0.45	0.35	0.35	0.45	0.23	0.35
SAT/mono-SAT PPE	0.02	0.78	0.95	0.78	0.35	0.08	0.95	1.00	0.23	0.45	0.45	0.13	0.13	0.02	0.08	0.68	0.35	0.23	0.23	0.35	0.52	0.23
PUFA-PPE	0.23	0.68	0.45	0.68	0.08	0.08	0.45	0.23	1.00	0.95	0.52	0.13	0.13	0.23	0.08	0.68	0.45	0.68	0.68	0.45	0.08	0.23
SAT/mono-SAT PC	0.45	0.95	0.78	0.95	0.68	0.78	0.78	0.45	0.95	1.00	0.45	0.52	0.52	0.45	0.78	0.52	0.68	0.35	0.35	0.68	0.68	0.35
PUFA-PC	0.45	0.35	0.23	0.35	0.78	0.68	0.23	0.45	0.52	0.45	1.00	0.95	0.95	0.45	0.68	0.52	0.68	0.95	0.95	0.68	0.68	0.08
SAT/mono-SAT EPC	0.13	0.23	0.95	0.23	0.08	0.08	0.95	0.13	0.13	0.52	0.95	1.00	0.02	0.13	0.08	0.68	0.08	0.13	0.13	0.08	0.45	0.45
PUFA-EPC	0.13	0.23	0.95	0.23	0.08	0.08	0.95	0.13	0.13	0.52	0.95	0.02	1.00	0.13	0.08	0.68	0.08	0.13	0.13	0.08	0.45	0.45
PG	0.02	0.78	0.95	0.78	0.35	0.08	0.95	0.02	0.23	0.45	0.45	0.13	0.13	1.00	0.08	0.68	0.35	0.23	0.23	0.35	0.52	0.23
22:6-PL	0.08	0.52	0.78	0.52	0.13	0.02	0.78	0.08	0.08	0.78	0.68	0.08	0.08	0.08	1.00	0.95	0.23	0.35	0.35	0.23	0.23	0.35
20:4-LBPA	0.68	0.45	0.08	0.45	0.95	0.95	0.08	0.68	0.68	0.52	0.52	0.68	0.68	0.68	0.95	1.00	0.35	0.13	0.13	0.35	0.35	0.68
VLC-SM & VLC-Cer	0.35	0.08	0.45	0.08	0.23	0.23	0.45	0.35	0.45	0.68	0.68	0.08	0.08	0.35	0.23	0.35	1.00	0.08	0.08	0.02	0.78	0.95
GalCer	0.23	0.23	0.35	0.23	0.45	0.35	0.35	0.23	0.68	0.35	0.95	0.13	0.13	0.23	0.35	0.13	0.08	1.00	0.02	0.08	0.95	0.78
LacCer	0.23	0.23	0.35	0.23	0.45	0.35	0.35	0.23	0.68	0.35	0.95	0.13	0.13	0.23	0.35	0.13	0.08	0.02	1.00	0.08	0.95	0.78
SL	0.35	0.08	0.45	0.08	0.23	0.23	0.45	0.35	0.45	0.68	0.68	0.08	0.08	0.35	0.23	0.35	0.02	0.08	0.08	1.00	0.78	0.95
GM3	0.52	0.95	0.23	0.95	0.23	0.23	0.23	0.52	0.08	0.68	0.68	0.45	0.45	0.52	0.23	0.35	0.78	0.95	0.95	0.78	1.00	0.45
Sph	0.23	0.78	0.35	0.78	0.35	0.35	0.35	0.23	0.23	0.35	0.08	0.45	0.45	0.23	0.35	0.68	0.95	0.78	0.78	0.95	0.45	1.00
